# MethyLasso: a segmentation approach to analyze DNA methylation patterns and identify differentially methylated regions from whole-genome datasets

**DOI:** 10.1093/nar/gkae880

**Published:** 2024-10-18

**Authors:** Delphine Balaramane, Yannick G Spill, Michaël Weber, Anaïs Flore Bardet

**Affiliations:** CNRS UMR7242 Biotechnologie et signalisation cellulaire, Université de Strasbourg, 300 Bd Sébastien Brant, 67412 Illkirch Cedex, France; Institut de Génétique et de Biologie Moléculaire et Cellulaire (IGBMC), 1 rue Laurent Fries, 67404 Illkirch Cedex, France; Centre National de Recherche scientifique (CNRS) UMR7104, 1 rue Laurent Fries, 67404 Illkirch Cedex, France; Université de Strasbourg, 1 rue Laurent Fries, 67404 Illkirch Cedex, France; Institut National de santé et de Recherche Médicale (INSERM) U1258, 1 rue Laurent Fries, 67404 Illkirch Cedex, France; CNRS UMR7242 Biotechnologie et signalisation cellulaire, Université de Strasbourg, 300 Bd Sébastien Brant, 67412 Illkirch Cedex, France; CNRS UMR7242 Biotechnologie et signalisation cellulaire, Université de Strasbourg, 300 Bd Sébastien Brant, 67412 Illkirch Cedex, France; CNRS UMR7242 Biotechnologie et signalisation cellulaire, Université de Strasbourg, 300 Bd Sébastien Brant, 67412 Illkirch Cedex, France; Institut de Génétique et de Biologie Moléculaire et Cellulaire (IGBMC), 1 rue Laurent Fries, 67404 Illkirch Cedex, France; Centre National de Recherche scientifique (CNRS) UMR7104, 1 rue Laurent Fries, 67404 Illkirch Cedex, France; Université de Strasbourg, 1 rue Laurent Fries, 67404 Illkirch Cedex, France; Institut National de santé et de Recherche Médicale (INSERM) U1258, 1 rue Laurent Fries, 67404 Illkirch Cedex, France

## Abstract

DNA methylation is an epigenetic mark involved in the regulation of gene expression, and patterns of DNA methylation anticorrelate with chromatin accessibility and transcription factor binding. DNA methylation can be profiled at the single cytosine resolution in the whole genome and has been performed in many cell types and conditions. Computational approaches are then essential to study DNA methylation patterns in a single condition or capture dynamic changes of DNA methylation levels across conditions. Toward this goal, we developed MethyLasso, a new approach to segment DNA methylation data. We use it as an all-in-one tool to perform the identification of low-methylated regions, unmethylated regions, DNA methylation valleys and partially methylated domains in a single condition as well as differentially methylated regions between two conditions. We performed a rigorous benchmarking comparing existing approaches by evaluating the agreement of the regions across tools, their number, size, level of DNA methylation, boundaries, cytosine–guanine content and coverage using several real datasets as well as the sensitivity and precision of the approaches using simulated data and show that MethyLasso performs best overall. MethyLasso is freely available at https://github.com/bardetlab/methylasso.

## Graphical Abstract

**Graphical Abstract:**
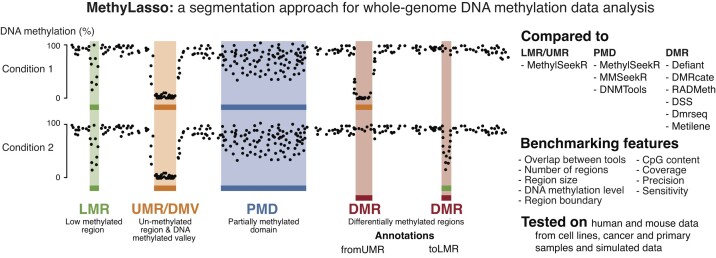

## Introduction

DNA methylation is an epigenetic mark that consists in the addition of a methyl group to cytosines mainly in the context of cytosine–guanine (CpG) dinucleotides. It is involved in the repression of gene expression as it is able to block transcription factors from binding to DNA (1,2). Patterns of DNA methylation anticorrelate with transcription factor binding where most of the inactive genome is fully methylated and active regulatory regions bound by transcription factors are low- or unmethylated (LMRs or UMRs) (3,4). Large UMRs that often mark developmental genes were termed as DNA methylation valleys (DMVs) (5,6) and partially methylated domains (PMDs) have been described as large domains with heterogeneous methylation levels in immortalized cell lines and cancer samples (7). DNA methylation is a reversible mark and demethylation was shown to be induced by transcription factor binding (3,8,9). DNA methylation patterns are therefore dynamic notably during cellular differentiation throughout development and disease conditions such as cancer.

Several experimental approaches have been developed to profile DNA methylation patterns (10). Bisulfite conversion (11) and more recently enzymatic conversion (12) followed by high-throughput sequencing can be applied to profile whole-genome DNA methylation at single cytosine resolution (Bis-seq or EM-seq, respectively). Computational approaches are then essential to study DNA methylation patterns in a single condition or capture dynamic changes of DNA methylation levels across conditions.

The identification of hypomethylated regions in a single condition is of great interest as it can be used to predict active regulatory regions bound by transcription factors. Originally, methylation levels were segmented using a hidden Markov model and distinct regions were described as UMRs with DNA methylation between 0% and 10%, LMRs between 10% and 50% and fully methylated regions (FMRs) above 50% (3). MethylSeekR (4) is the most widely used tool to identify LMRs and UMRs. In order to circumvent the uncertainty of methylation levels due to the low sequencing coverage of individual CpGs, MethylSeekR smoothes methylation levels over three consecutive CpGs with a minimal coverage of five reads. It then identifies hypomethylated regions as stretches of consecutive CpGs with methylation below 50%. Since it expects LMRs to be located at CpG-poor regions and UMRs to be found at CpG-rich regions called CpG islands, MethylSeekR uses CpG content as a threshold to further classify regions as LMRs or UMRs. This threshold (default to 30 CpGs) has to be set manually after visualizing the CpG content plot generated. False discovery rate (FDR) is computed for LMRs using shuffled CpG methylation levels. However, the use of a CpG threshold is not pertinent for all organisms. Furthermore, some completely unmethylated regions could have few CpGs and some low methylated regions could have high CpG density (3). On a more technical note, MethylSeekR only calls LMRs and UMRs for each dataset individually and is not able to integrate replicates from the same condition.

PMDs were originally described as regions with average methylation levels below 70% in merged bins of 10 kb (7). They have been compared in a large number of samples and shown to be shared across cell types (13,14). MethylSeekR (4) was the first tool to identify PMDs in order not to call UMRs and LMRs within PMDs as they could be artifacts. PMDs are identified using a two-state hidden Markov model using a beta-binomial distribution in sliding windows of 100 consecutive CpGs and classified according to the methylation levels. A plot showing the distribution of posterior mean of *α* (one of the two shape parameters of the beta distribution used in the MethylSeekR model) helps the user manually decide if the sample contains PMDs or not if it has a bimodal or long-tailed distribution with an important proportion of *α* > 1. More recently, multimodel PMD SeekR (MMSeekR) (15) was developed as an extension of the MethylSeekR hidden Markov model. It combines the MethylSeekR *α* score to a Pearson correlation coefficient between the DMVs and a neural network score of single CpGs that takes the local sequence context into account in non-overlapping windows of 201 CpGs. It enables to also identify PMDs with extreme hypomethylation. However, MMSeekR can only be used on the human genome for which the neural network score is available. DNMTools (16) was developed to free the user from manual inspection to decide whether the data contains PMDs or not before using the tool. It also applies a two-state hidden Markov model using a beta-binomial distribution based on their previous tool MethPipe (17). It then further refines the boundaries of the regions to obtain a dynamic bin size and filters out small PMDs by measuring an FDR comparing the size of the regions with shuffled ones. On a more technical note, all the tools call PMDs for each dataset individually and are not able to integrate replicates from the same condition.

The identification of differentially methylated regions (DMRs) to compare conditions is one of the main analyses performed when investigating DNA methylation patterns. Many tools with various approaches have been developed for whole-genome methylation datasets and several reviews have compared the different approaches conceptually (18) and benchmarked them (19–21). The DMRs identified by the different approaches have a very limited overlap [example in (19)], which makes it difficult for the user to choose one. Some approaches rely on fixed or predefined windows and some first rely on the identification of single differentially methylated CpGs (DMCs) and then aggregates them into DMRs (18), which may lead to missed DMRs or low precision of DMR boundaries. The DMRs identified by the different approaches also differ in terms of number, size, level of DNA methylation differences, CpG content and coverage. On a more technical note, some approaches can only be used if several replicates for each condition are available, which are not always available due to the cost of generating genome-wide methylation profiles.

In this study, we tested six of the most used and recent ones. One of the first approaches developed to identify DMRs is BSmooth (22), which applies local likelihood smoothing to overcome potential biases due to low coverage. It requires replicates to call DMCs using a *t*-test and groups the ones above a specific threshold into DMRs. RADmeth (23) is based on a beta-binomial regression approach. It calls significant DMCs using a log-likelihood ratio test and neighboring DMCs are combined using a weighted *Z*-test to obtain DMRs. DSS (24) is based on a Bayesian hierarchical model based on the beta-binomial distribution. It can also smooth the methylation values, calls significant DMCs using a Wald test and groups them as DMRs using thresholds such as *P*-value, minimum length and minimum number of CpGs. Defiant (25) employs an approach where the sample’s variance is weighted based on coverage. It calls significant DMCs using a Welch’s *t*-test if several replicates are available or a Fisher’s exact test otherwise and uses a weighted Welch expansion to identify DMRs. Dmrseq (26), which now replaces BSmooth, also performs smoothing and uses the same beta-binomial approach as used in DSS to call significant DMCs. It then uses a continuous autoregressive correlation to identify DMRs and controls for FDR using the Benjamini and Hochberg procedure. DMRcate (27), that was first developed for methylation array data, was recently adapted for whole-genome Bis-seq data and uses limma to generate per-CpG *t*-statistics and kernel smoothing and groups them into DMRs using an FDR threshold. Only few approaches define DMRs directly based on the changes of DNA methylation differences without any assumption about the data distribution. Metilene (28) performs a first pre-segmentation of the genome and then a circular binary segmentation approach is used to iteratively reduce the size of the regions to maximize the mean DNA methylation difference. Segmentation stops when a minimum of CpGs is reached or when the two-dimensional Kolmogorov–Smirnov *P*-value does not improve. Adjusted *P*-values are also provided as well as *P*-values from a Mann–Whitney *U*-test.

Here, we present MethyLasso, a new segmentation approach to analyze DNA methylation patterns and identify DMRs from whole-genome datasets. MethyLasso models DNA methylation data using a regression framework known as generalized additive model. It relies on the fused lasso to estimate regions in which the methylation is constant. The strength of this statistical framework, which we leveraged successfully in the past in a different genomic application (29), is that it does not require binning of data, i.e. grouping and averaging of methylation values on contiguous sites, prior to performing the analysis. Indeed, binning of data raises several issues, first of which is the proper size of the bins. If they are too small, statistical models can quickly be overwhelmed by the low signal-to-noise ratio in the data. If they are too large, the resulting estimates risk to agglomerate neighboring but unrelated genomic features. The fused lasso statistical regression method solves this dilemma and permits to identify regions where methylation can be considered constant.

We apply this approach to the methylation values in a single condition to identify hypomethylated regions, i.e. LMRs, UMRs and DMVs, solely based on the methylation levels of the segments independent of CpG content. We apply it on the variation of methylation values in a single condition to identify large domains with heterogeneous methylation levels, i.e. PMDs. Finally, we apply it on the differences of methylation levels between two conditions to identify DMRs and annotate them using the previous calls, i.e. LMRs, UMRs, DMVs or PMDs.

We compare MethyLasso with other methods that were shown to perform best in the literature and perform a rigorous benchmarking investigating the regions’ overlap, number, size, methylation levels or changes, boundaries, CpG content and coverage using several real datasets. We further measured the sensitivity and precision of the DMR approaches using simulated data. Using all those metrics, we show that MethyLasso performs best overall.

## Materials and methods

### Input data format

MethyLasso runs on whole-genome DNA methylation data (e.g. Bis-seq or EM-seq) where DNA methylation is measured for each cytosine in the genome, which can then be summed for each CpG. The data take the form of a matrix containing for each C or CpG position in the genome (chromosome, start and end), the percent of methylated sequences (meth) out of the total number of sequences covering this position (cov). This can alternatively be calculated from the number of methylated Cs (mC) and the number of unmethylated Cs (uC). By default, data in a bismark output format (30) are used (chromosome, start, end, percent_methylation, count_methylated and count_unmethylated) but other formats can also be specified (see MethyLasso README). MethyLasso only keeps by default positions covered by at least five reads (program argument -c).

### MethyLasso general framework

MethyLasso models DNA methylation data using a nonparametric regression framework known as a generalized additive model. It relies on the fused lasso method (31) to segment the genome by estimating regions in which methylation is constant. This model is adapted from the one applied for Hi-C data in binless normalization (29). For a whole-genome DNA methylation dataset from a single condition with *N* CpGs, the resulting data take the form of a *N*-dimensional vector of the observed methylated fraction *y* = *M*/*C* from methylated counts *M* (program argument -mC), and total sequencing coverage *C* (program argument --cov). *y* is given a normal likelihood with unknown true methylation fraction *β*. A one-dimensional fused lasso prior is then placed on *β*, resulting in a minus log posterior proportional to the L1-penalized weighted least squares target. *λ*_2_ corresponds to the strength of the fusion.


\begin{equation*}\mathop \sum \limits_{i = 1}^N {{c}_i}{{\left( {{{y}_i} - {{{\mathrm{\beta }}}_i}} \right)}^2} + {{\lambda }_2}\mathop \sum \limits_{i = 2}^N \left| {{{{\mathrm{\beta }}}_i} - {{{\mathrm{\beta }}}_{i - 1}}} \right|.\end{equation*}

In the above formula, *i* is the index of each CpG as they appear along the genome. We chose a normal likelihood out of practical considerations, as a binomial likelihood, also adapted to this context, would require an iterated reweighted least squares approximation, which rendered computational costs prohibitive in early attempts with marginal quality benefits. The computational gains and rationale are similar to what prompted the development of ‘fast binless’ compared with the original binless approach, as described in (29). The L1 norm is the fused lasso term and is a computationally tractable surrogate for the nonconvex L0 regularization problem. A common alternative is to use the L2 norm, which would result in smoothed estimates, and does not result in sharp decision boundaries as required here.

**Figure 1.**
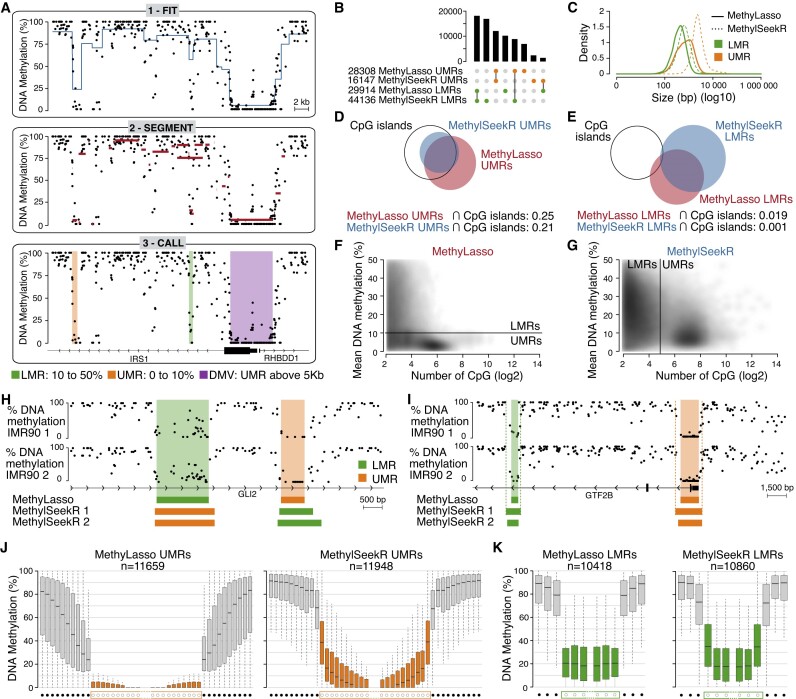
Identification of LMRs, UMRs and DMVs. (**A**) Genome browser view of the MethyLasso segmentation of the DNA methylation data in IMR90 cells. 1. Fit the model to the data. 2. Segment the data. 3. Call the LMRs, UMRs and DMVs. (**B**) Upset plot of the number and overlap of UMRs and LMRs called by MethyLasso and MethylSeekR. MethylSeekR regions for replicate 1 are shown. (**C**) Histogram of the size of the LMRs and UMRs called by MethyLasso and MethylSeekR. (**D**) Venn diagram showing the overlap of CpG islands with UMRs from MethyLasso and MethylSeekR. (**E**) Venn diagram showing the overlap of CpG islands with LMRs from MethyLasso and MethylSeekR. (**F**) Density plot of the number of CpGs versus mean DNA methylation in each region from MethyLasso. The black line represents the MethyLasso threshold between LMRs (top panel) and UMRs (bottom panel). (**G**) Density plot of the number of CpGs versus DNA methylation in each region from MethylSeekR. The black line represents the MethylSeekR threshold between LMRs (left panel) and UMRs (right panel). (**H**) Genome browser view of an example of different annotations of LMRs and UMRs between MethyLasso and MethylSeekR. (**I**) Genome browser view of an example of differences in boundaries between MethyLasso and MethylSeekR LMR and UMR regions. (**J**) Boxplot of the DNA methylation at individual CpGs at UMR boundaries both called by MethyLasso and MethylSeekR. Gray boxes correspond to the 10 CpGs upstream and downstream of the UMRs. Orange boxes correspond to the 10 CpGs at the beginning or the end of the UMRs. (**K**) Boxplot of the DNA methylation at individual CpGs at LMR boundaries both called by MethyLasso and MethylSeekR. Gray boxes correspond to the three CpGs upstream and downstream of the LMRs. Green boxes correspond to the three CpGs at the beginning or the end of the LMRs.

To help understand the formula, we can refer to Figure 1A1 in which each dot corresponds to the observed methylation fraction *y_i_*, while the fused lasso estimates of the true methylation fraction at each of these positions, *β_i_*, have been connected and formed the blue line. The coverage *c_i_* for each CpG is not shown in the figure, but the left sum in the formula (called the least squares term) indicates that the positions with better coverage will influence the estimation proportionally more. During the estimation procedure, the formula is to be minimized by adjusting the value of the *β_i_*. The least squares term encourages *β_i_* values to be close to *y_i_* for each *i*. The second sum (called the penalty term) encourages neighboring *β_i_* values to be close. In fact, owing to the mathematical properties of the absolute value function, they are encouraged to be equal. The optimum for *β_i_* therefore has to respond to two potentially conflicting objectives: being close to *y_i_* on one hand and being equal to *β_i_* − 1 and *β_i_* + 1 on other hand. The optimal solution for the whole *β* vector of values is therefore piecewise constant: contiguous values of *β_i_* are equal, until the local methylation context provided by successive *y_i_* becomes so different that the values of the *β_i_* ‘jump’ to another value. The number of plateaus and associated jumps is set implicitly by the fusion term ${\mathrm{\lambda }}2$. Higher values of ${\mathrm{\lambda }}2$ will increase the penalty and therefore decrease the number of jumps.

We model one set of coefficients ${\mathrm{\beta }}$ by condition. In the case of multiple replicates per condition, or multiple conditions, a design matrix *X* that maps replicates to conditions is built and the expectation of *y* is then *Xβ*. When replicates are available, MethyLasso accounts for variability in terms of coverage of the different replicates but does not integrate variability across replicates in the regions output scores. To integrate replicates, we add the numbers of methylated Cs and unmethylated Cs for each CpG to recompute the coverages *c_i_* and observed methylation values *y_i_* for each condition. We then model each chromosome independently. In the presence of two conditions, the design matrix is chosen such that one set of ${\mathrm{\beta }}$ coefficients represents the methylation of the reference condition, while the remaining sets of coefficients represent the methylation difference of the remaining condition to the reference. The resulting maximum posterior estimate *β* is a piecewise constant function, which is then used as a basis to construct a segmentation of the methylation data. For example, for two conditions KO and WT with two replicates *A* and *B* each, the formula for a single chromosome generalizes as follows:


\begin{eqnarray*} &&\mathop \sum \limits_{r = A,B} \mathop \sum \limits_{i = 1}^N c_i^{{\rm WT},r}{{\left( {y_i^{{\rm WT},r} - {\mathrm{\beta }}_i^{{\rm ref}}} \right)}^2}\nonumber\\ &&+ \mathop \sum \limits_{r = A,B} \mathop \sum \limits_{i = 1}^N c_i^{{\rm KO},r}{{\left( {y_i^{{\rm KO},r} - {\mathrm{\beta }}_i^{{\rm ref}} - {\mathrm{\beta }}_i^{{\rm diff}}} \right)}^2}\\ &&+ {{{\mathrm{\lambda }}}_2}\mathop \sum \limits_{c = {\rm ref},{\rm diff}} \mathop \sum \limits_{i = 2}^N \left| {{\mathrm{\beta }}_i^c - {\mathrm{\beta }}_{i - 1}^c} \right|.\end{eqnarray*}

### Identification of LMRs, UMRs and DMVs

#### Segmentation of the genome

For each single condition (possibly with multiple replicates), MethyLasso performs a fused lasso segmentation of the methylation values with ${{\lambda }_2}$ set to 25 to be able to identify small regions of constant methylation (smaller ${{\lambda }_2}$ would identify smaller regions and larger ${{\lambda }_2}$ larger ones). We obtain a vector of methylated fractions *β*, with one coefficient per CpG. This vector is piecewise constant and forms a first crude segmentation of the methylome. Segments are split when they contain gaps in between two CpGs of 500 bp or more. Their borders are adjusted to start and end on a CpG. Then, for each segment, we compute the number of CpGs, mean methylation and standard deviation.

#### Identification of LMRs and UMRs

We first define admissible segments if they have a minimum size of 10 bp and a minimum of four CpGs (program argument -n). Among admissible segments, we define segments of interest as those whose methylation is smaller than that of its nearest admissible neighbors (on both sides). We then define flanking segments as admissible segments with a minimum size of 300 bp (excluding small artifacts), which are immediate neighbors to a segment of interest but are not segments of interest themselves. Candidate LMRs are defined as segments of interest that are separated from their flanking segments by at least 2 standard deviations (clear dip in the signal), whose mean methylation is below 0.5 fitting the original definition (3) and whose flanking segments are no further away than 5000 bp (which would be outliers). Candidate LMRs that simultaneously have a CpG count below 10 and flanking segments with a methylation below 0.5 are not retained in the final LMR call as they are supported by only few CpGs and only have a small dip in the signal. Candidate LMRs that have a mean methylation below 0.1 and simultaneously whose standard deviation is smaller than 0.1 are instead called UMRs as they fit better to the original UMR definition (3). Remaining candidate LMRs constitute the final LMR call. Finally, among segments of interest with a mean methylation below 0.1 and standard deviation below 0.1 that were not part of the candidate LMR set, we declare those that have a size above 500 bp and >30 CpGs/kb as additional UMRs as they might represent large UMRs known to be enriched at CpG islands with no clear dip in the signal if the flanking regions look like shores (32) known not to have intermediate methylation levels. LMRs or UMRs that overlap a DMV (see ahead) are not reported as they are part of the DMVs and LMRs that overlap a PMD are not reported as they cannot be clearly distinguished from PMDs with high standard deviation (see ahead).

#### Identification of DMVs

We further group segments whose mean methylation is continuously below 0.1 as long as they are not separated by a large gap of 5 kb, merge them to UMRs and define them as DMVs if their size is ≥5 kb and the average distance between CpGs is ≤500 bp fitting the original definition (5,6). We then recompute their number of CpGs, mean methylation and standard deviation.

#### Output files

MethyLasso outputs one file per condition containing the genomic coordinates and characteristics of the regions (chromosome, start, end, number of CpGs, mean methylation, standard deviation and annotation as LMRs and UMRs including DMVs). It also generates plots showing histograms of the reads sequencing depth and CpG methylation from positions with at least five reads (see MethyLasso README) (Supplementary Figure S1A and B).

### Identification of PMDs

#### Segmentation of the genome

For each single condition (possibly with multiple replicates), MethyLasso performs a fused lasso segmentation of the standard deviation with ${{\lambda }_2}$ set to 1000 to identify large regions where methylation varies widely (smaller ${{\lambda }_2}$ would identify smaller regions and larger ${{\lambda }_2}$ larger ones). Segments are split when they contain gaps in between two CpGs of 5 kb or more or when they contain a UMR or DMV. Then, for each segment, we compute the number of CpGs, mean methylation and standard deviation.

#### Identification of PMDs

We first define admissible segments if they have a size >5 kb, a minimum of four CpGs (program argument -n) and an average distance between CpGs <500 bp (to exclude outlier regions that are very large with few CpGs). PMDs are then defined as admissible segments with a mean methylation below 70% (program argument -m) fitting to the original definition (7) and a standard deviation above 0.15. Contiguous PMDs are then merged into a single region. We then recompute their number of CpGs, mean methylation and standard deviation.

#### Output files

MethyLasso outputs one file per condition containing the genomic coordinates and characteristics of the regions (chromosome, start, end, number of CpGs, mean methylation, standard deviation and annotation as PMDs). It also generates a smoothed scatterplot showing the methylation level compared to the standard deviation of all segments (Supplementary Figure S1C).

### Identification of DMRs

#### Segmentation of the genome

In presence of two conditions (possibly with multiple replicates), the statistical model is designed such that one set of coefficients represents the methylation of the reference condition, while the remaining sets of coefficients represent the methylation difference of the remaining condition to the reference. Each set of coefficients is obtained by a one-dimensional fused lasso as described with ${{\lambda }_2}$ set to 25 to be able to identify small regions, and yields a segmentation of the methylation differences between a condition and the reference. Then, for each segment, we compute the number of CpGs, mean methylation difference and standard deviation.

#### Identification of DMRs

We group neighboring segments when their mean methylation difference is below the threshold, 10% by default (program argument -d). Their borders are adjusted so that they start and end on a CpG. We then recompute their number of CpGs, mean methylation difference and standard deviation. We also compute a coverage score for each DMR as the percent of CpGs covered in all samples (conditions and replicates) and discard DMRs with a CpG coverage score below 70% (program argument -r) to make sure that the mean methylation difference of the region is well supported by data from most CpGs within the region. Finally, we calculate the Wilcoxon *P*-value for each DMR by comparing the methylation at each CpG in the two conditions. FDR is controlled using the Benjamini–Hochberg procedure on the *P*-values. We retain DMRs with a minimum of four CpGs (program argument -n), a methylation difference ≥10% (program argument -d) and a *P*-value ≤0.05 (program argument -p). Alternatively, DMRs can also be selected according to the FDR threshold (program argument -q).

#### Annotation of DMRs using LMRs, UMRs, DMVs and PMDs

If LMRs, UMRs, DMVs and PMDs were defined in each condition, DMRs will be annotated according to those annotations. DMRs are annotated in each condition by the regions with which it overlaps most if any and at least 10% for small LMRs, UMRs and DMVs and 50% for large PMDs. Change of annotation is then indicated as from the reference condition to the other, e.g. LMRtoUMR, (nothing)toPMD and fromDMV(to nothing).

#### Output files

MethyLasso outputs one file containing the genomic coordinates and characteristics of the regions (chromosome, start, end, number of CpGs in each condition, CpG coverage score, mean methylation in each condition, mean methylation difference, *P*-value, FDR and annotation). It also generates a smoothed scatterplot showing the methylation level of the DMRs in the reference condition compared with the other condition (Supplementary Figure S1D).

Most of the parameters set above were optimized using the datasets described in this work and can be modified either directly as arguments of MethyLasso.R or in the functions of the R files provided on GitHub (see ‘Data availability’ section).

### MethyLasso benchmarking

#### DNA methylation datasets

We used whole-genome Bis-seq datasets publicly available in the NCBI Gene Expression Omnibus (GEO) repository from human embryonic stem cells (ESH1) and fetal lung fibroblasts cells (IMR90) (7) (GEO accession numbers GSM429321, GSM429322, GSM429323, GSM432685 and GSM432686 for ESH1 one replicate, GSM432687, GSM432688 and GSM432689 for IMR90 replicate 1 and GSM432690, GSM432691 and GSM432692 for IMR90 replicate 2), primary human colorectal cancer and normal cells (33) (GEO accession numbers GSM1204465 and GSM1204466, respectively, with three replicates each) and mouse hematopoietic stem cells (HSCs) and multipotent progenitor populations (MPPs) (34) (GEO accession numbers GSM1274424, GSM1274425 and GSM1274426 for HSC three replicates and GSM1274433, GSM1274434 and GSM1274435 for MPP three replicates). We also downloaded 167 datasets analyzed in (13) from three databases, namely Blueprint, NIH Roadmap Epigenomics and the International Human Epigenome Consortium Data Portal. The list of the samples used is available from the supplementary files (GEO accession number GSE113405).

#### Data processing

Raw sequencing reads were trimmed using trim_galore (version 0.6.4 options -q 20 --stringency 2) (http://www.bioinformatics.babraham.ac.uk/projects/trim_galore/) and mapped to the human genome reference hg38 or the mouse mm10 using bismark (version 0.22.1) (30). Nonconverted and duplicated reads were filtered out using filter_non_conversion –percentage_cutoff 50 –minimum_count 5 and deduplicate_bismark. Methylation levels were extracted using bismark_methylation_extractor. Methylation was summarized per CpG by overlapping the methylation levels per cytosine with CpGs of the reference genome using a custom script.

#### Simulated DMRs

We used whole-genome Bis-seq data containing simulated DMRs from Metilene to evaluate the performance of MethyLasso and other methods. They simulated DMRs with two different backgrounds (homogeneous background 1 and heterogeneous background 2), each with four subsets, from the largest methylation difference to the smallest. Each subset has 10 replicates. For precision and sensitivity, 95% confidence intervals were calculated in R using the binom.test function from the ‘stats’ package, and for the *F*1 score, the delta method was used to approximate the confidence interval. Data are available from http://www.bioinf.uni-leipzig.de/Software/metilene/Downloads/.

#### Comparison with other methods

For the identification of LMRs and UMRs, we compared MethyLasso with MethylSeekR (version 3.6.1) (4). MethylSeekR was executed with default settings. Since MethylSeekR does not identify DMVs, we called our DMVs as UMRs when comparing UMRs. In order to compare methylation levels in LMRs and UMRs from the two different programs, we recomputed them by first summing the counts from the replicates for each condition to calculate the methylation level per CpG and then calculating the mean methylation in the region.

For the identification of PMDs, we compared MethyLasso with three other programs, namely MethylSeekR (4), MMSeekR (version 1.0) (15) and DNMTools (version 1.4.2) (16). We used the default parameters for each program to call PMDs. PMDs called by MethylSeekR for the comparison of 167 datasets from (13) were directly downloaded from the supplementary files (GEO accession number GSE113405). For MMSeekR and DNMTools, we called PMDs for each sample individually and calculated the mean DNA methylation in the domains. Note that for MethylSeekR and MMSeekR, the PMDs were called in order to compare the results but most 167 samples did not have PMDs after manual inspection of the *α* or *M* value plots, and in those cases, those tools should not have been applied to call PMDs.

For the identification of DMRs, we compared MethyLasso with six other programs, namely Defiant (1.1.9) (25), Dmrseq (1.6.0) (26), DSS (2.34.0) (24), RadMeth (23), DMRcate (2.14.0) (27) and Metilene (0.2-8) (28). We used those programs with default setting except for a minimum coverage of five reads per CpG, a minimum of four CpGs and at least 10% methylation change to call DMRs. DSS was run using the smoothing option as recommend for whole-genome Bis-seq data. We also ran Dmrseq, RadMeth, Metilene and DMRcate with more relaxed thresholds in order to obtain the best 150 000 DMRs for colorectal cancer versus normal cells, best 270 000 DMRs for ESH1 versus IMR90 data and best 5000 for HSC versus MPP, in order to consider a maximum of regions for all tools while minimizing the number of tools for which we used nondefault relaxed parameters. In order to compare methylation differences in DMRs from the different programs, we recomputed them by first summing the counts from the replicates for each condition to calculate the methylation level per CpG, then calculating the difference between two conditions and finally calculating the mean difference in the region. The DMRs identified by the different methods are available to load on the UCSC Genome Browser (My Data/Track Hubs) using the following URL: https://g-948214.d2cf88.03c0.data.globus.org/hub_methylasso.txt.

#### Computational setup

Benchmarking was performed on a server using an Intel Xeon Silver CPU (10 cores, 20 threads, 2.2 GHz and 96 GB RAM) and MethyLasso can also run on a MacBook Air with a 1.6 GHz Intel Core i5 processor and 8 GB of memory. For example, the analyses on our server using the primary human colorectal cancer and normal cells data took 29 min and 47 GB of RAM using 10 threads and 2h 20 min and 53 GB of RAM using one thread.

#### Data analyses

All genomic data analyses were performed using bash scripts using the command awk and bedtools (35) and R for plots and statistics. The upset plots we generated using the intervene R package (36).

### Software implementation and availability

MethyLasso’s segmentation code is written in C++ and the regions’ identification code in R. MethyLasso’s code and manual as well as the code developed to benchmark the methods are available on GitHub at https://github.com/bardetlab/methylasso.

## Results

### Identification of LMRs, UMRs and DMVs

MethyLasso is a method that first analyzes DNA methylation patterns in each experimental condition independently. It relies on a fused lasso approach to segment the genome by estimating regions in which the methylation is constant (see ‘Materials and methods’ section). It fits the model to DNA methylation data, segments the data and calls different regions such as LMRs (10–50% methylation), UMRs (0–10% methylation) or DMVs (UMRs larger than 5 kb) according to specific thresholds (Figure 1A; see ‘Materials and methods’ section). One advantage over MethylSeekR is that MethyLasso can integrate replicates when available to call each set of regions only once per condition. On data from human IMR90 fibroblast cells (7), MethyLasso identified 29 914 LMRs and 28 308 UMRs, including 240 DMVs, whereas MethylSeekR identified more LMRs but less UMRs in both replicates independently (see Figure 1B and Supplementary Figure S2 for other datasets). As expected, UMRs often located at CpG island promoters are larger than LMRs (Figure 1C–E, and Supplementary Figure S3). We observe that LMRs are more often identified by only one of the approaches than UMRs (Figure 1B, D and E), which can be explained by the fact that UMRs have more CpGs with DNA methylation levels constrained within 0–10%, whereas LMRs can have a broader middle range of methylation levels (10–50%) with higher variance, so are expected to be more difficult to identify. When looking at the number of CpGs versus the mean methylation level in each of the regions (UMRs + LMRs), we observe that they cluster as expected: regions with high CpG density such as CpG islands are mostly unmethylated and regions with low CpG density have higher methylation levels (Figure 1F and G, and Supplementary Figure S2). However, we decided not to include the CpG density as a main parameter to identify UMRs and LMRs as some regions with few CpGs can be completely unmethylated (Figure 1F, bottom left corner). Therefore, MethyLasso only sets the threshold between LMRs and UMRs according to their DNA methylation level matching the original definition (3) from 0% to 10% for UMRs and from 10% to 50% for LMRs independently of their CpG density (Figure 1F, black line). In contrast, MethylSeekR sets the threshold between LMRs and UMRs according to their CpG density (30 CpGs for IMR90 data) (Figure 1G, black line) and therefore some UMRs predicted by MethylSeekR are not completely unmethylated, whereas some LMRs are (Figure 1G). Since MethylSeekR UMRs correspond to regions dense in CpGs, it also explains why they are larger than MethyLasso UMRs (Figure 1C). Therefore, because of the difference of definition of the regions, 8913 MethyLasso UMRs are identified as LMRs by MethylSeekR and 1481 LMRs as UMRs (Figure 1B and H, and Supplementary Figure S2). Even when both programs agree on calling UMRs or LMRs, their region boundaries still differ and even depend on the replicate for MethylSeekR (Figure 1I). To evaluate this systematically, we compared the methylation of CpGs within UMRs or LMRs to the CpGs immediately upstream or downstream (Figure 1J and K). For UMRs, we observe that both MethyLasso and MethylSeekR show significant shifts of DNA methylation levels between neighboring CpGs across boundaries. However, MethyLasso identifies smaller regions (Figure 1C) that are completely unmethylated, whereas MethylSeekR includes surrounding CpGs that have higher methylation levels without being fully unmethylated (Figure 1J) that might represent CpG island shores (32). For LMRs, we observe that CpGs across boundaries have better shifts in DNA methylation level for MethyLasso than for MethylSeekR and that CpGs within MethyLasso LMRs have more homogeneous DNA methylation levels (Figure 1K). Similar findings were obtained in several other human or mouse Bis-seq datasets (Supplementary Figure S4).

### Identification of PMDs

**Figure 2.**
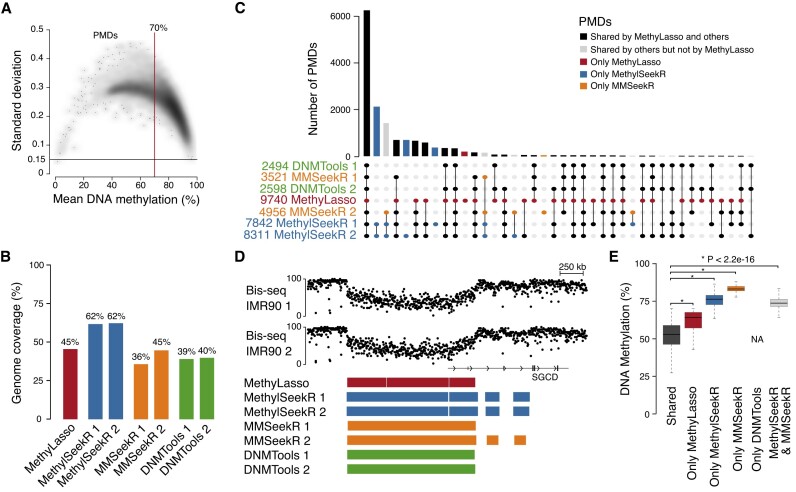
Identification of PMDs. (**A**) Scatterplot showing for all MethyLasso segments their mean DNA methylation versus standard deviation. Segments with mean methylation below 70% and standard deviation above 0.15 are called as PMDs. (**B**) Barplot showing the percent of the genome covered by PMDs called by different methods in two replicates in IMR90 cells. (**C**) Upset plot of the number and overlap of PMDs from the different methods. (**D**) Genome browser view of an example of PMDs shared by all methods or only found by MethylSeekR and MMSeekR. Region from hg38 at chr5:154800000–156500000. (**E**) Boxplot of the mean DNA methylation of the PMDs shared by all methods or only found by one or two methods. Corresponding Wilcoxon *P*-value.

MethyLasso identifies PMDs by segmenting the genome based on DNA methylation variation into large regions above 5 kb with mean methylation levels below 70% by default and variation of DNA methylation, i.e. standard deviation above 0.15 (Figure 2A; see ‘Materials and methods’ section). In contrast to MethylSeekR (4) and MMSeekR (15), MethyLasso does not require a preliminary manual inspection to define if the data contain PMDs or not (Supplementary Figure S5A–D). When calling PMDs on data from human embryonic stem cells ESH1 (7) expected not to have any PMDs (Supplementary Figure S5A and E), MethyLasso indeed only identified 61 PMDs. In human IMR90 fibroblast data, expected to have PMDs (Figure 2A, and Supplementary Figure S5B and F), MethyLasso identified 9740 PMDs covering 45% of the genome, which is similar for MMSeekR and DNMTools and higher for MethylSeekR (Figure 2B). One advantage of MethyLasso compared with others is that it can integrate replicates when available to call PMDs only once for the condition. We observe that the majority of PMDs are identified by all methods (43%) but a substantial fraction is only identified by MethylSeekR and/or MMSeekR (which is an improvement of MethylSeekR) and few by MethyLasso only and none by DNMTools only (Figure 2C and D). However, PMDs only identified by MethylSeekR, MMSeekR or both have much higher level of DNA methylation above 70%, which do not qualify as PMDs according to MethyLasso thresholds (Figure 2D and E). Similar results were obtained for data in human colon cancer compared with healthy samples (33) where healthy datasets are not expected to have PMDs and cancer datasets are (Supplementary Figure S5C, D, G, H, I, J, K, N, O and P). Additionally, we observe for the healthy data that the few PMDs only identified by DNMTools surprisingly have very low DNA methylation levels, which are identified as LMRs/UMRs/DMVs by MethyLasso (Supplementary Figure S5L and M).

PMDs have been studied in a large number of samples and found to be largely shared (13,14). To test this, we reanalyzed 167 methylomes mostly from primary cells where PMDs were identified using MethylSeekR from (13). We see that MethyLasso identified only few samples as containing PMDs with 21 having >5% of their genome covered by PMDs (Supplementary Figure S6A) and all PMDs having methylation levels below our 70% threshold (Supplementary Figure S6B). This is a stark difference with MethylSeekR results, which identified a consistent 50% of the genome being covered by PMDs [Supplementary Figure S6C; PMD calls from (13)]. MMSeekR, derived from MethylSeekR, agreed with this (Supplementary Figure S6E), whereas DNMTools agreed with MethyLasso (Supplementary Figure S6G). However, in samples where MethylSeekR and MMSeekR identified PMDs and MethyLasso and DNMTools did not, we observed that the least MethyLasso identified PMDs (order of the *x*-axis), the most MethylSeekR and MMSeekR identified PMDs with high methylation level (above 85% for 105 out of 167 samples) (Supplementary Figure S6D and F). This is due to the fact that MethylSeekR (or MMSeekR) should only be used on samples showing evidence of PMDs in the distribution of posterior mean of *α* (Supplementary Figure S6I and J), and if not, the two-state hidden Markov model is forced to identify those two states even though they both have very high methylation levels. Further, if we investigate the proportion of MethylSeekR PMDs below or above the 70% methylation threshold applied by MethyLasso, we observe that, as expected, true PMDs below 70% are more likely to be shared across samples (Supplementary Figure S6K). For DNMTools, again, the few PMDs identified in those samples had low methylation levels (Supplementary Figure S6H) and would be called as LMRs, UMRs or DMVs by MethyLasso.

In summary, we show that MethyLasso is an accurate tool to identify PMDs with reduced methylation in any dataset without prior knowledge of the presence of PMDs.

### Identification of DMRs

**Figure 3.**
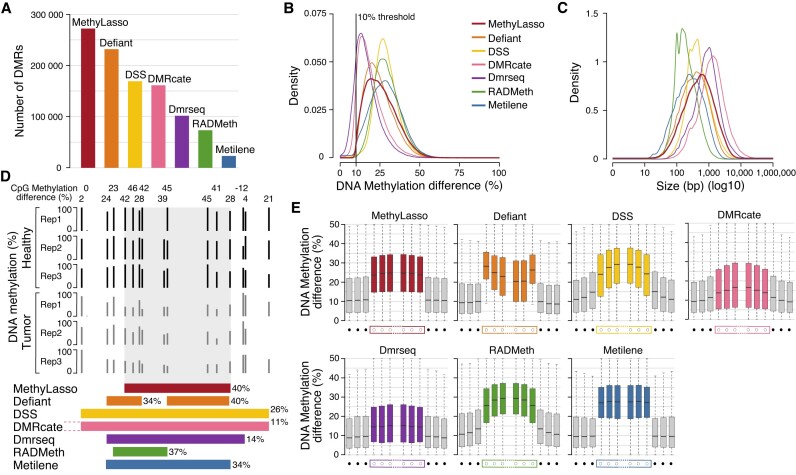
Identification of DMRs. (**A**) Barplot of the number of DMRs between healthy and colon cancer cells identified by the different methods. (**B**) Histogram of the absolute DNA methylation difference in the best 150 000 DMRs called by the different methods. (**C**) Histogram of the size of the best 150 000 DMRs called by the different methods. (**D**) Genome browser view of an example of DMRs with different region boundaries found by all methods. DMR from DMRcate extend much upstream of the region shown (chr5:72417868–72419158). Methylation difference is shown in percent for all individual CpGs and for DMRs from each method. Region from hg38 at chr5:72418800–72419200. (**E**) Boxplot of the difference of DNA methylation at individual CpGs at the best 150 000 DMR region boundaries called by the different methods. Gray boxes correspond to the three CpGs upstream and downstream of the DMRs. Colored boxes correspond to the three CpGs at the beginning or the end of the DMRs. Comparisons between CpGs located outside (gray) versus in (red) DMRs are significant for all approaches. Wilcoxon *P*-value<2.2e−16.

MethyLasso can also identify DMRs in DNA methylation data from different conditions. It fits the model to DNA methylation differences across conditions, segments the data and calls DMRs according to specific thresholds such as by default a mean methylation difference above 10% and a minimum of four CpGs (see ‘Materials and methods’ section). It also annotates the DMRs using the previous segmentation from condition 1 to condition 2, e.g. LMRtoUMR, (nothing)toPMD and fromDMV(to nothing). To evaluate the performance of MethyLasso in identifying DMRs, we compared it with six other approaches, namely Defiant (25), DSS (24), DMRcate (27), Dmrseq (26), Radmeth (23) and Metilene (28), using Bis-seq datasets from human colon cancer compared with healthy samples (33). MethyLasso identified the most DMRs (272 123) in cancer versus healthy samples (Figure 3A). Defiant, DSS and DMRcate also identified several hundreds of thousands of DMRs, whereas Dmrseq, Radmeth and Metilene identified much fewer DMRs (only 23 141 for Metilene) (Figure 3A). In order to compare the different tools, we focused our analyses on the best 150 000 DMRs from each method using relaxed threshold when fewer regions were identified by default. When looking at the DMRs’ DNA methylation differences, Dmrseq and DMRcate mostly identified DMRs with small differences, MethyLasso and Defiant identified more DMRs with small differences close to the 10% threshold but also some with big differences, whereas DSS, Radmeth and Metilene identified mostly DMRs with bigger differences (Figure 3B). When looking at the DMR’s size, MethyLasso, Defiant and Metilene identified DMRs with a wide range of sizes, whereas other approaches have a more limited range with Radmeth DMRs being short, DSS DMRs having an intermediate size and Dmrseq and DMRcate DMRs being large (Figure 3C). We also evaluated the DMRs’ boundaries as we noticed that the DMRs identified by the different tools have varying boundaries that can extend to neighboring CpGs with lower methylation differences (Figure 3D). To evaluate this systematically, we compared the three first and last CpGs within DMRs with the three CpGs immediately upstream or downstream outside DMRs. We observed that MethyLasso DMRs have sharp boundaries of DNA methylation differences compared with the neighboring CpGs (Figure 3E) and homogeneous levels of methylation differences within DMRs. Defiant DMRs also have sharp boundaries but the median methylation difference within DMRs is less homogeneous. In contrast, the methylation differences at the DMR boundaries of DSS, DMRcate and Radmeth have a bell shape and the CpGs just outside their DMRs still have methylation differences higher than 10% (our threshold), indicating that their boundaries are less accurate. DMRcate and Dmrseq DMRs only have small shifts and CpGs in DMRs have lower DNA methylation differences indicating that their boundaries are not accurate. This might be due to the fact that it smoothes the data before calling DMRs and identifies mainly large DMRs. For Metilene, which is also a segmentation-based approach, its DMR boundaries are well defined (Figure 3E). Importantly, we obtained similar results when identifying DMRs in completely different datasets: human ESH1 versus IMR90 cells (7) with big DNA methylation differences, or mouse HSCs versus MPPs (34) that show small changes in DNA methylation (Supplementary Figure S7). Altogether, these analyses show that MethyLasso identifies a higher number of DMRs with clear boundaries compared with existing DMR tools.

### Consistency of DMRs from different approaches

**Figure 4.**
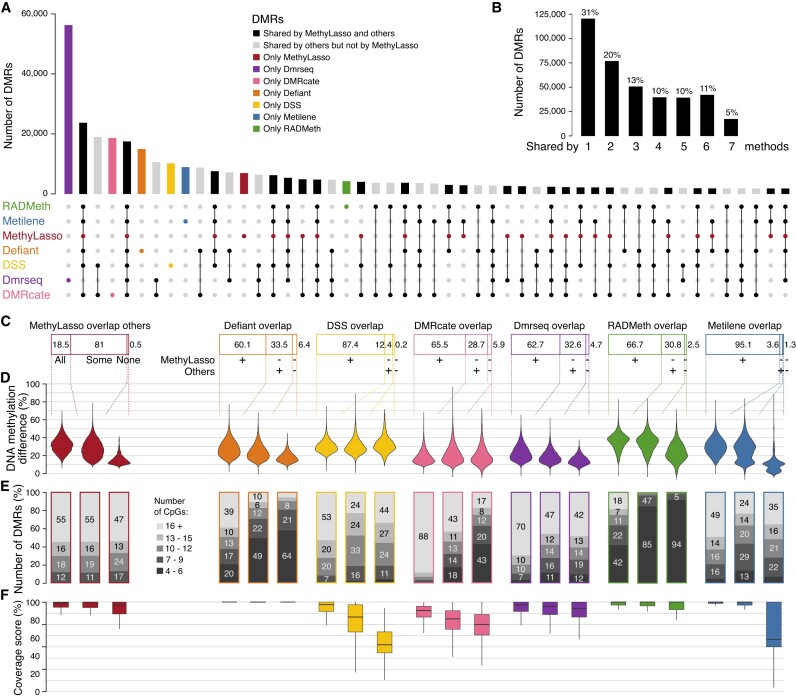
Consistency between DMRs from different approaches. (**A**) Upset plot of the overlap of best 150 000 DMRs called by the different methods merged into 386 580 regions. Bars corresponding to DMRs identified by MethyLasso are represented in red. (**B**) Barplot summarizing the upset plot by summing the number of regions identified by all, some or none of the methods. (**C**) Cumulative barplot showing the percent of best 50 000 MethyLasso DMRs overlapping with all others 150 000 DMRs, some others or none of the other methods. For other methods, percent of their best 50 000 DMRs overlapping with 150 000 DMRs from MethyLasso (+), not MethyLasso but others (−/+) or not MethyLasso nor others (−/−). (**D**) Absolute DNA methylation difference in the DMRs from the categories in panel (C). (**E**) Cumulative barplot showing the number of CpGs in the DMRs from the categories in panel (C). (**F**) Boxplot showing the coverage of CpGs in the DMRs from the categories in panel (C).

Next, we investigated the consistency of MethyLasso DMRs compared with the other methods. To compare the DMRs from the different approaches, we first analyzed the overlap between the best 150 000 DMRs identified by any approach merged into 386 580 regions. Most are shared by at least two approaches (69%) but only 5% are shared by all and a substantial fraction is found by only one method (31%) (Figure 4A and B). As expected, the fraction of DMRs shared by all and therefore likely to be true increased with an increasing methylation difference, with a strong improvement starting at 20% methylation difference (Supplementary Figure S8). However, the evaluation of the DMR’s overlap might be underestimated since some might be shared but with ranks slightly below our threshold (37). Therefore, we asked whether the very best 50 000 DMRs from one approach were identified by the best 150 000 DMRs of the other approaches. Out of the best 50 000 DMRs identified by MethyLasso, 18.5% were found by all other approaches but 81% were found by some others and only 0.5% were not found by any other approach (see Figure 4C, red, and Supplementary Figure S9 for examples and all data available for visualization in a UCSC hub; see ‘Materials and methods’ section). When looking at the difference in methylation of those DMRs, we observed that the DMRs found by MethyLasso and others had bigger methylation differences than the ones found by MethyLasso alone (Figure 4D, red). This might be explained by the fact that DMRs with small changes in DNA methylation are more difficult to identify. Nevertheless, DMRs found by MethyLasso alone had a similar CpG content than the DMRs found by MethyLasso and others (including some with many CpGs) (Figure 4E, red). This suggests that MethyLasso DMRs are likely true. When looking at the best 50 000 DMRs identified by others compared with the whole 150 000 sets, most were also identified by MethyLasso (Figure 4C; 95.1% for Metilene, 87.4% for DSS, 66.7% for RADmeth, 65.5 for DMRcate, 62.7% for Dmrseq and 60.1% for Defiant). We then analyzed the DMRs found by these other methods but not MethyLasso. Defiant DMRs not found by MethyLasso had low methylation differences (Figure 4D, orange) and were enriched in DMRs with a small number of CpGs (between 4 and 6; Figure 4E, orange), which could be artifacts. DSS DMRs not found by MethyLasso do not have smaller methylation differences (Figure 4D, yellow) and lower CpG numbers (Figure 4E, yellow), but we observed that they had a low coverage of their CpGs below the 70% coverage threshold set by MethyLasso (see Figure 4F, yellow, and Supplementary Figure S9 for examples and all data available for visualization in a UCSC hub; see ‘Materials and methods’ section). Most DMRs from DMRcate are large regions (Figure 3C, pink) with low methylation differences (Figure 4D, pink) but some of the ones not identified by MethyLasso have a low number of CpGs (Figure 4E, pink) and a low coverage of their CpGs (Figure 4F, pink). Dmrseq DMRs are also large regions (Figure 3C, purple) and have overall low methylation differences that are even lower when not found by MethyLasso (Figure 4D, purple) but still have similar number of CpGs (Figure 4E, purple). Radmeth DMRs that are small in size (Figure 3C, green) have overall higher methylation difference but DMRs not found by any others have lower methylation differences (Figure 4D, green).

However, most of Radmeth DMRs not found by MethyLasso have a very low number of CpGs (Figure 4E, green), which could be artifacts. Finally, only very few Metilene DMRs were not found by MethyLasso (Figure 4E, blue). Some have a low coverage of its CpGs (Figure 4F, blue) and 59 have no coverage at all for one of the conditions (see Supplementary Figure S9 for examples), which is due to the fact that Metilene imputes missing data. Importantly, we obtained similar results when identifying DMRs in completely different cell lines, namely human ESH1 versus IMR90 cells (7) with bigger DNA methylation changes or in differentiating cells, namely mouse HSC versus MPP cells (34) with small changes in DNA methylation (Supplementary Figures S10 and S11). Altogether, these analyses show that MethyLasso identifies DMRs that are also identified by others, and that DMRs from others not identified by MethyLasso tend to have features that could come from artifacts.

### Sensitivity and precision of the approaches using simulated DMRs

**Figure 5.**
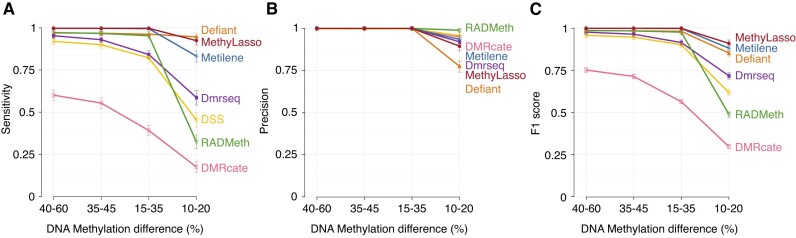
Sensitivity and precision of the method using simulated DMRs. Simulated DMRs in different bins of DNA methylation difference from Metilene using a heterogeneous background. (**A**) Sensitivity or recall with 95% confidence intervals of the predictions measured as the number of true positives among all simulated. (**B**) Precision with 95% confidence intervals of the predictions measured as the number of true positives among all predicted. (**C**) *F*1 score or accuracy with delta approximation of the confidence intervals of the predictions measured using both the sensitivity and precision.

To evaluate the performance of MethyLasso at identifying DMRs and compare it with other approaches and since no ground truth is available using real data, we called DMRs on simulated data with varying levels of DNA methylation differences (data from Metilene) (see ‘Materials and methods’ section). We first investigated the sensitivity of the approach, which evaluates the fraction of simulated DMRs that are correctly identified or true positive rate (Figure 5A). For DMRs with large methylation differences (40–60%), which should be the easiest to identify, only MethyLasso and Metilene were able to identify all simulated DMRs (sensitivity at 1). For DMRs with small methylation differences (10–20%), which should be more difficult to identify, Defiant and MethyLasso performed best followed by Metilene, whereas Dmrseq, DSS, Radmeth and DMRcate performed worse. We then investigated the precision of the approach, which evaluates the fraction of predicted DMRs that were indeed simulated (Figure 5B). For DMRs with large methylation differences, all programs identified well the simulated DMRs. For DMRs with small methylation differences, all programs performed well with Radmeth being best closely followed by DSS, DMRcate, Metilene, Dmrseq and MethyLasso and Defiant being less precise. Finally, we calculated the *F*1 score, i.e. the harmonic mean of the sensitivity and precision and identified MethyLasso and Metilene as performing perfectly on DMRs with large differences and MethyLasso followed by Metilene and then Defiant on DMRs with small differences (Figure 5C). Performances and ranking are similar when a different set of simulated DMRs are used (Supplementary Figure S12). In summary, we conclude that MethyLasso outperforms existing tools on the analysis of simulated DMRs.

## Discussion

MethyLasso is a new approach to analyze DNA methylation patterns from whole-genome DNA methylation datasets. It mostly differs from other tools as it is based on a segmentation of the DNA methylation patterns, which enables it to identify precise region boundaries.

MethyLasso can analyze data from each condition independently and can integrate replicates to identify LMRs, UMRs and DMVs by performing a segmentation of the DNA methylation levels. It differs from MethylSeekR mainly by defining regions of interest based on their DNA methylation level matching their definition (LMR or UMR) rather than based on their CpG content (Figure 1F and G). Additionally, we show that MethyLasso performs better at defining the region’s boundaries since it does not smooth methylation levels (Figure 1J and K). For UMRs, MethylSeekR tends to include neighboring regions with higher methylation level that might represent CpG island shores (32). Since MethyLasso identifies regions with constant methylation level, it excludes UMR shores, which also results in lower mean methylation of the region that are therefore less likely to be annotated as LMR instead of UMR.

MethyLasso can identify PMDs by performing a segmentation of the DNA methylation variation. It focuses on large regions with heterogeneous DNA methylation levels and does not identify MethylSeekR and MMSeekR PMDs with high methylation levels (Figure 2D and E, and Supplementary Figure S6). Although those might have slightly lower methylation levels than the rest of the genome, we decided to keep the original definition of PMDs as having a high methylation variance with mean methylation below 70%. Importantly, this enables not to rely on the manual inspection of the results to decide to call PMDs or not (Supplementary Figures S5A–D and S6I). However, the definition of PMDs as well as their biological significance remains elusive and we give the user the opportunity to visualize the methylation and standard deviation of the MethyLasso segments and change the default methylation threshold if necessary. DNMTools PMDs largely agree with MethyLasso but identify few LMRs that MethyLasso identifies as LMRs, UMRs or DMVs.

MethyLasso can also be applied to compare DNA methylation levels across two conditions by performing a segmentation of the DNA methylation differences that defines DMRs based on the overall methylation patterns rather than grouping individual CpGs. MethyLasso can integrate replicates when available and annotates the DMRs using the previous LMR, UMR, DMV and PMD calls. This could be useful, for example, if one wishes to remove DMRs located in PMDs, which represent a large fraction of the healthy versus colon cancer DMRs (61%) and of the ESH1 versus IMR90 DMRs (75%). When comparing different tools, the DMRs identified have a limited overlap (Figure 4A and B) indicating that the identification of DMRs is a challenging task. As expected, DMRs with bigger DNA methylation changes have a higher overlap than DMRs with small changes (Supplementary Figure S8).

MethyLasso identifies a large number of DMRs, some with big DNA methylation changes and more as expected with small changes close to the cutoff (Figure 3A and B). MethyLasso DMRs have a wide range of sizes showing that it can adapt well to the size of potential DMRs (Figure 3C). The boundaries of the MethyLasso DMRs are well defined at locations with sharp transitions of DNA methylation changes with all CpGs within DMRs having stable high levels of DNA methylation differences and CpGs just outside having stable levels close to the 10% cutoff (Figure 3E). Ninety-nine percent of the best MethyLasso DMRs are identified by at least one other method indicating that they are most likely true positives (Figure 4C). The few DMRs not identified by any of the others have small methylation changes and are therefore more difficult to identify. Between 95% (Metilene) and 60% (Defiant) of the best DMRs from other methods are identified by MethyLasso. Most of the ones missed by MethyLasso have small methylation changes. DMRs identified by Defiant, RADmeth and DMRcate and not by MethyLasso are mainly DMRs containing few CpGs (4–6). Some DMRs identified by DSS, DMRcate and Metilene but missed by MethyLasso still have high levels of methylation changes but come from regions that are not well covered (DSS) or where data were imputed (Metilene). Finally, using simulated DMRs with different bins of DNA methylation change, MethyLasso is the approach with the best overall results especially in term of sensitivity where it identifies best the simulated DMRs with both high and low methylation changes (Figure 5).

Defiant DMRs have a wide range of DNA methylation differences and size and good region boundaries. However, they have the least overlap with MethyLasso ones (60%), the most not found by any others (6.4%) and those have only few CpGs (Figure 4C and E), all of which indicate that they could be artifacts. On simulated data, its sensitivity is high but it surprisingly misses DMRs with big methylation changes and its precision is lower than all others as it identifies DMRs that were not simulated (Figure 5B).

DSS tends to identify DMRs with bigger methylation changes but less well-defined region boundaries that might be due to smoothing (Figure 3B and E). It also includes regions with low CpG coverage that might bias the methylation levels and represent false positives (Figure 4F, and Supplementary Figure S9). On simulated data, DSS has a poor sensitivity as it misses DMRs that have been simulated even with big DNA methylation changes (Figure 5A).

DMRcate identifies large DMRs and does not adapt well to short regions of DNA methylation changes resulting in DMRs with small methylation changes (Figure 3B and C). Its DMR boundaries are less well defined, which might be due to smoothing (Figure 3E). On simulated data, DMRcate shows the worse sensitivity as it misses DMRs that have been simulated even with big DNA methylation changes (Figure 5A).

Dmrseq also identifies large DMRs with small methylation changes and not well-defined boundaries, which might be due to smoothing (Figure 3B, C and E). On simulated data, Dmrseq shows a poor sensitivity as it misses DMRs that have been simulated even with big DNA methylation changes (Figure 5A). Additionally, Dmrseq can only be applied if replicates are available.

RADmeth identifies a low number of DMRs with small size and therefore less well-defined region boundaries (Figure 3B, C and E). On simulated data, RADmeth shows to have a poor sensitivity as it misses DMRs that have been simulated even with big DNA methylation changes (Figure 5).

Metilene only identifies very few DMRs (10 times less than MethyLasso) with big methylation changes even though the threshold is set to 10% (Figure 3A and B). Like MethyLasso, Metilene applies a segmentation approach resulting in well-defined region boundaries. The overlap between Metilene and MethyLasso DMRs is very high and the few DMRs only identified by Metilene are mostly due to the fact that it imputes missing data, generating DMRs with artificially high methylation changes (Figure 4F). On simulated data that were generated by the authors of Metilene, where it might have an advantage, Metilene performs worse than MethyLasso in terms of sensitivity and slightly better in terms of precision (Figure 5A and B).

In conclusion, MethyLasso is an all-in-one approach that performs a robust segmentation to analyze DNA methylation patterns either in a single condition to identify LMRs, UMRs, DMVs and PMDs or by comparing conditions to identify DMRs. We conducted an extensive benchmark to show that MethyLasso performs best compared with state-of-the-art tools. Since DNA methylation levels anticorrelate with chromatin accessibility, the identification of LMRs, UMRs and DMRs is a powerful approach to predict active regulatory regions bound by transcription factors that regulate gene expression.

## Supplementary Material

gkae880_Supplemental_File

## Data Availability

MethyLasso is available on github from https://github.com/bardetlab/methylasso. MethyLasso's original code is also available at https://zenodo.org/records/13829420.

## Notes

Present address: Yannick G. Spill, European Food Safety Authority (EFSA), Via Carlo Magno, 1A, 43126 Parma, Italy.

## References

[1] Bird A. DNA methylation patterns and epigenetic memory. Genes Dev. 2002; 16:6–21. 11782440 10.1101/gad.947102

[2] Domcke S. , Bardet A.F., Ginno P.A., Hartl D., Burger L., Schübeler D. Competition between DNA methylation and transcription factors determines binding of NRF1. Nature. 2015; 528:575–579. 26675734 10.1038/nature16462

[3] Stadler M.B. , Murr R., Burger L., Ivanek R., Lienert F., Schöler A., van Nimwegen E., Wirbelauer C., Oakeley E.J., Gaidatzis D. et al. DNA-binding factors shape the mouse methylome at distal regulatory regions. Nature. 2011; 480:490–495. 22170606 10.1038/nature10716

[4] Burger L. , Gaidatzis D., Schübeler D., Stadler M.B. Identification of active regulatory regions from DNA methylation data. Nucleic Acids Res. 2013; 41:e155. 23828043 10.1093/nar/gkt599 PMC3763559

[5] Xie W. , Schultz M.D., Lister R., Hou Z., Rajagopal N., Ray P., Whitaker J.W., Tian S., Hawkins R.D., Leung D. et al. Epigenomic analysis of multilineage differentiation of human embryonic stem cells. Cell. 2013; 153:1134–1148. 23664764 10.1016/j.cell.2013.04.022 PMC3786220

[6] Jeong M. , Sun D., Luo M., Huang Y., Challen G.A., Rodriguez B., Zhang X., Chavez L., Wang H., Hannah R. et al. Large conserved domains of low DNA methylation maintained by Dnmt3a. Nat. Genet. 2014; 46:17–23. 24270360 10.1038/ng.2836 PMC3920905

[7] Lister R. , Pelizzola M., Dowen R.H., Hawkins R.D., Hon G., Tonti-Filippini J., Nery J.R., Lee L., Ye Z., Ngo Q.-M. et al. Human DNA methylomes at base resolution show widespread epigenomic differences. Nature. 2009; 462:315–322. 19829295 10.1038/nature08514 PMC2857523

[8] Pastor W.A. , Aravind L., Rao A. TETonic shift: biological roles of TET proteins in DNA demethylation and transcription. Nat. Rev. Mol. Cell Biol. 2013; 14:341–356. 23698584 10.1038/nrm3589 PMC3804139

[9] Detilleux D. , Spill Y.G., Balaramane D., Weber M., Bardet A.F. Pan-cancer predictions of transcription factors mediating aberrant DNA methylation. Epigenetics Chromatin. 2022; 15:1–16. 35331302 10.1186/s13072-022-00443-w PMC8944071

[10] Plongthongkum N. , Diep D.H., Zhang K. Advances in the profiling of DNA modifications: cytosine methylation and beyond. Nat. Rev. Genet. 2014; 15:647–661. 25159599 10.1038/nrg3772

[11] Cokus S.J. , Feng S., Zhang X., Chen Z., Merriman B., Haudenschild C.D., Pradhan S., Nelson S.F., Pellegrini M., Jacobsen S.E. Shotgun bisulphite sequencing of the *Arabidopsis* genome reveals DNA methylation patterning. Nature. 2008; 452:215–219. 18278030 10.1038/nature06745 PMC2377394

[12] Vaisvila R. , Ponnaluri V.K.C., Sun Z., Langhorst B.W., Saleh L., Guan S., Dai N., Campbell M.A., Sexton B.S., Marks K. et al. Enzymatic methyl sequencing detects DNA methylation at single-base resolution from picograms of DNA. Genome Res. 2021; 31:1280–1289. 34140313 10.1101/gr.266551.120 PMC8256858

[13] Salhab A. , Nordström K., Gasparoni G., Kattler K., Ebert P., Ramirez F., Arrigoni L., Müller F., Polansky J.K., Cadenas C. et al. A comprehensive analysis of 195 DNA methylomes reveals shared and cell-specific features of partially methylated domains. Genome Biol. 2018; 19:1–13. 30266094 10.1186/s13059-018-1510-5 PMC6161375

[14] Zhou W. , Dinh H.Q., Ramjan Z., Weisenberger D.J., Nicolet C.M., Shen H., Laird P.W., Berman B.P. DNA methylation loss in late-replicating domains is linked to mitotic cell division. Nat. Genet. 2018; 50:591–602. 29610480 10.1038/s41588-018-0073-4 PMC5893360

[15] Zheng Y. , Ziman B., Ho A.S., Sinha U.K., Xu L.-Y., Li E.-M., Koeffler H.P., Berman B.P., Lin D.-C. Comprehensive analyses of partially methylated domains and differentially methylated regions in esophageal cancer reveal both cell-type- and cancer-specific epigenetic regulation. Genome Biol. 2023; 24:193. 37620896 10.1186/s13059-023-03035-3 PMC10463844

[16] Decato B.E. , Qu J., Ji X., Wagenblast E., Knott S.R.V., Hannon G.J., Smith A.D. Characterization of universal features of partially methylated domains across tissues and species. Epigenetics Chromatin. 2020; 13:39. 33008446 10.1186/s13072-020-00363-7 PMC7532633

[17] Song Q. , Decato B., Hong E.E., Zhou M., Fang F., Qu J., Garvin T., Kessler M., Zhou J., Smith A.D. A reference methylome database and analysis pipeline to facilitate integrative and comparative epigenomics. PLoS One. 2013; 8:e81148. 24324667 10.1371/journal.pone.0081148 PMC3855694

[18] Shafi A. , Mitrea C., Nguyen T., Draghici S. A survey of the approaches for identifying differential methylation using bisulfite sequencing data. Brief. Bioinform. 2018; 19:737–753. 28334228 10.1093/bib/bbx013 PMC6171488

[19] Piao Y. , Xu W., Park K.H., Ryu K.H., Xiang R. Comprehensive evaluation of differential methylation analysis methods for bisulfite sequencing data. Int. J. Environ. Res. Public Health. 2021; 18:7975. 34360271 10.3390/ijerph18157975 PMC8345583

[20] Kreutz C. , Can N.S., Bruening R.S., Meyberg R., Mérai Z., Fernandez-Pozo N., Rensing S.A. A blind and independent benchmark study for detecting differentially methylated regions in plants. Bioinformatics. 2020; 36:3314–3321. 32181821 10.1093/bioinformatics/btaa191

[21] Peng X. , Luo H., Kong X., Wang J. Metrics for evaluating differentially methylated region sets predicted from BS-seq data. Brief. Bioinform. 2022; 23:bbab475. 34874989 10.1093/bib/bbab475

[22] Hansen K.D. , Langmead B., Irizarry R.A. BSmooth: from whole genome bisulfite sequencing reads to differentially methylated regions. Genome Biol. 2012; 13:R83. 23034175 10.1186/gb-2012-13-10-r83 PMC3491411

[23] Dolzhenko E. , Smith A.D. Using beta-binomial regression for high-precision differential methylation analysis in multifactor whole-genome bisulfite sequencing experiments. BMC Bioinform. 2014; 15:215. 10.1186/1471-2105-15-215 PMC4230021 24962134

[24] Park Y. , Wu H. Differential methylation analysis for BS-seq data under general experimental design. Bioinformatics. 2016; 32:1446–1453. 26819470 10.1093/bioinformatics/btw026 PMC12157722

[25] Condon D.E. , Tran P.V., Lien Y.-C., Schug J., Georgieff M.K., Simmons R.A., Won K.-J. Defiant: (DMRs: easy, fast, identification and ANnoTation) identifies differentially Methylated regions from iron-deficient rat hippocampus. BMC Bioinformatics. 2018; 19:31. 29402210 10.1186/s12859-018-2037-1 PMC5800085

[26] Korthauer K. , Chakraborty S., Benjamini Y., Irizarry R.A. Detection and accurate false discovery rate control of differentially methylated regions from whole genome bisulfite sequencing. Biostatistics. 2019; 20:367–383. 29481604 10.1093/biostatistics/kxy007 PMC6587918

[27] Peters T.J. , Buckley M.J., Chen Y., Smyth G.K., Goodnow C.C., Clark S.J. Calling differentially methylated regions from whole genome bisulphite sequencing with DMRcate. Nucleic Acids Res. 2021; 49:e109. 34320181 10.1093/nar/gkab637 PMC8565305

[28] Jühling F. , Kretzmer H., Bernhart S.H., Otto C., Stadler P.F., Hoffmann S. metilene: fast and sensitive calling of differentially methylated regions from bisulfite sequencing data. Genome Res. 2016; 26:256–262. 26631489 10.1101/gr.196394.115 PMC4728377

[29] Spill Y.G. , Castillo D., Vidal E., Marti-Renom M.A. Binless normalization of Hi-C data provides significant interaction and difference detection independent of resolution. Nat. Commun. 2019; 10:1938–1910. 31028255 10.1038/s41467-019-09907-2 PMC6486590

[30] Krueger F. , Andrews S.R. Bismark: a flexible aligner and methylation caller for Bisulfite-Seq applications. Bioinformatics. 2011; 27:1571–1572. 21493656 10.1093/bioinformatics/btr167 PMC3102221

[31] Tibshirani R. , Saunders M., Rosset S., Zhu J., Knight K. Sparsity and smoothness via the fused LASSO. J. R. Stat. Soc. Ser. B Stat. Methodol. 2005; 67:91–108.

[32] Irizarry R.A. , Ladd-Acosta C., Wen B., Wu Z., Montano C., Onyango P., Cui H., Gabo K., Rongione M., Webster M. et al. The human colon cancer methylome shows similar hypo- and hypermethylation at conserved tissue-specific CpG island shores. Nat. Genet. 2009; 41:178–186. 19151715 10.1038/ng.298 PMC2729128

[33] Hansen K.D. , Timp W., Bravo H.C., Sabunciyan S., Langmead B., McDonald O.G., Wen B., Wu H., Liu Y., Diep D. et al. Increased methylation variation in epigenetic domains across cancer types. Nat. Genet. 2011; 43:768–775. 21706001 10.1038/ng.865 PMC3145050

[34] Cabezas-Wallscheid N. , Klimmeck D., Hansson J., Lipka D.B., Reyes A., Wang Q., Weichenhan D., Lier A., von Paleske L., Renders S. et al. Identification of regulatory networks in HSCs and their immediate progeny via integrated proteome, transcriptome, and DNA methylome analysis. Cell Stem Cell. 2014; 15:507–522. 25158935 10.1016/j.stem.2014.07.005

[35] Quinlan A.R. , Hall I.M. BEDTools: a flexible suite of utilities for comparing genomic features. Bioinformatics. 2010; 26:841–842. 20110278 10.1093/bioinformatics/btq033 PMC2832824

[36] Khan A. , Mathelier A. Intervene: a tool for intersection and visualization of multiple gene or genomic region sets. BMC Bioinformatics. 2017; 18:287. 28569135 10.1186/s12859-017-1708-7 PMC5452382

[37] Bardet A.F. , He Q., Zeitlinger J., Stark A. A computational pipeline for comparative ChIP-seq analyses. Nat. Protoc. 2012; 7:45–61. 10.1038/nprot.2011.420 22179591

