# Supplementary material for: MethyLasso: a segmentation approach to analyze DNA methylation patterns and identify differentially methylated regions from whole-genome datasets
Source: Nucleic Acids Res. 2024 Oct 18;52(21):e98. doi: 10.1093/nar/gkae880 (PMC11602171; doi:10.1093/nar/gkae880)
Supplement: gkae880_Supplemental_File [file gkae880_supplemental_file.pdf]

MethyLasso: a segmentation approach to analyze DNA methylation patterns and identify differentially methylated regions from whole-genome datasets

Delphine Balaramane[1,2,3,4,5,†], Yannick G. Spill[1,†], Michaël Weber[1,*], Anaïs Flore Bardet[1,2,3,4,5,*]

SUPPLEMENTARY FIGURES

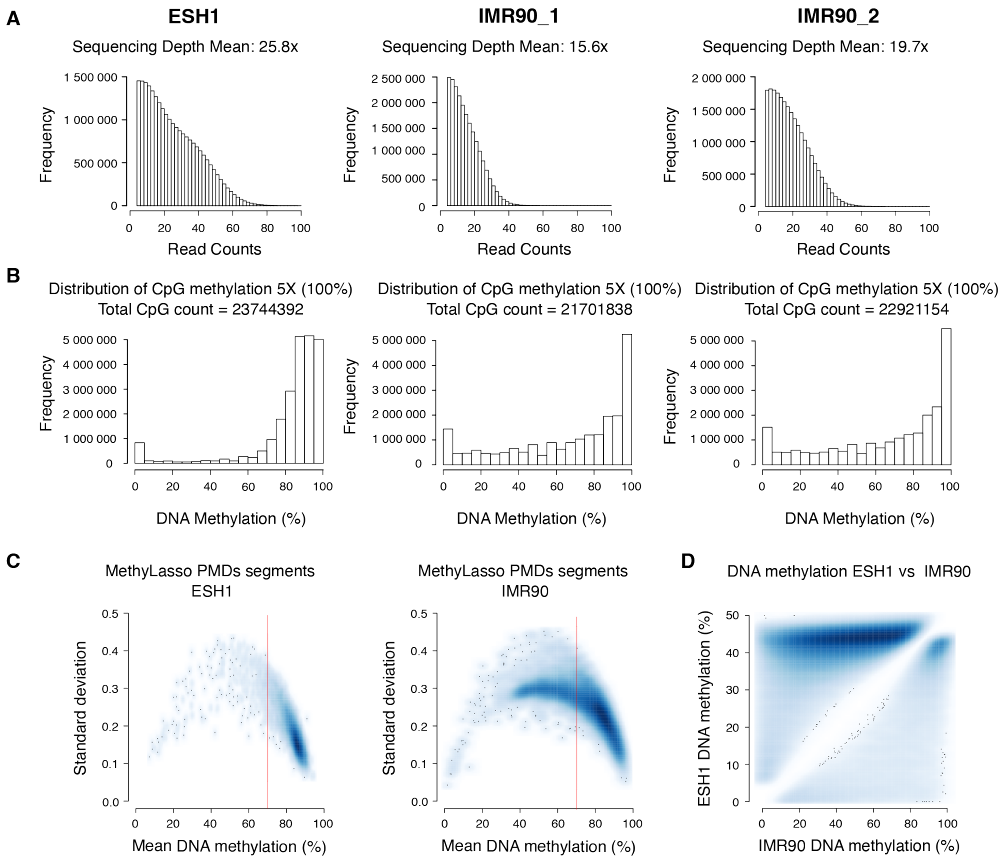

Supplementary Figure 1 - Example of output plots from MethyLasso

A. *Histograms of the distribution of the read sequencing depth for each sample.* ***B.*** *Histograms of the distribution of CpG methylation levels for each sample.* ***C.*** *Smoothed scatterplot of the methylation levels compared to the standard deviation of all PMD segments.* ***D.*** *Smoothed scatterplot the DMRs methylation levels in both conditions.*

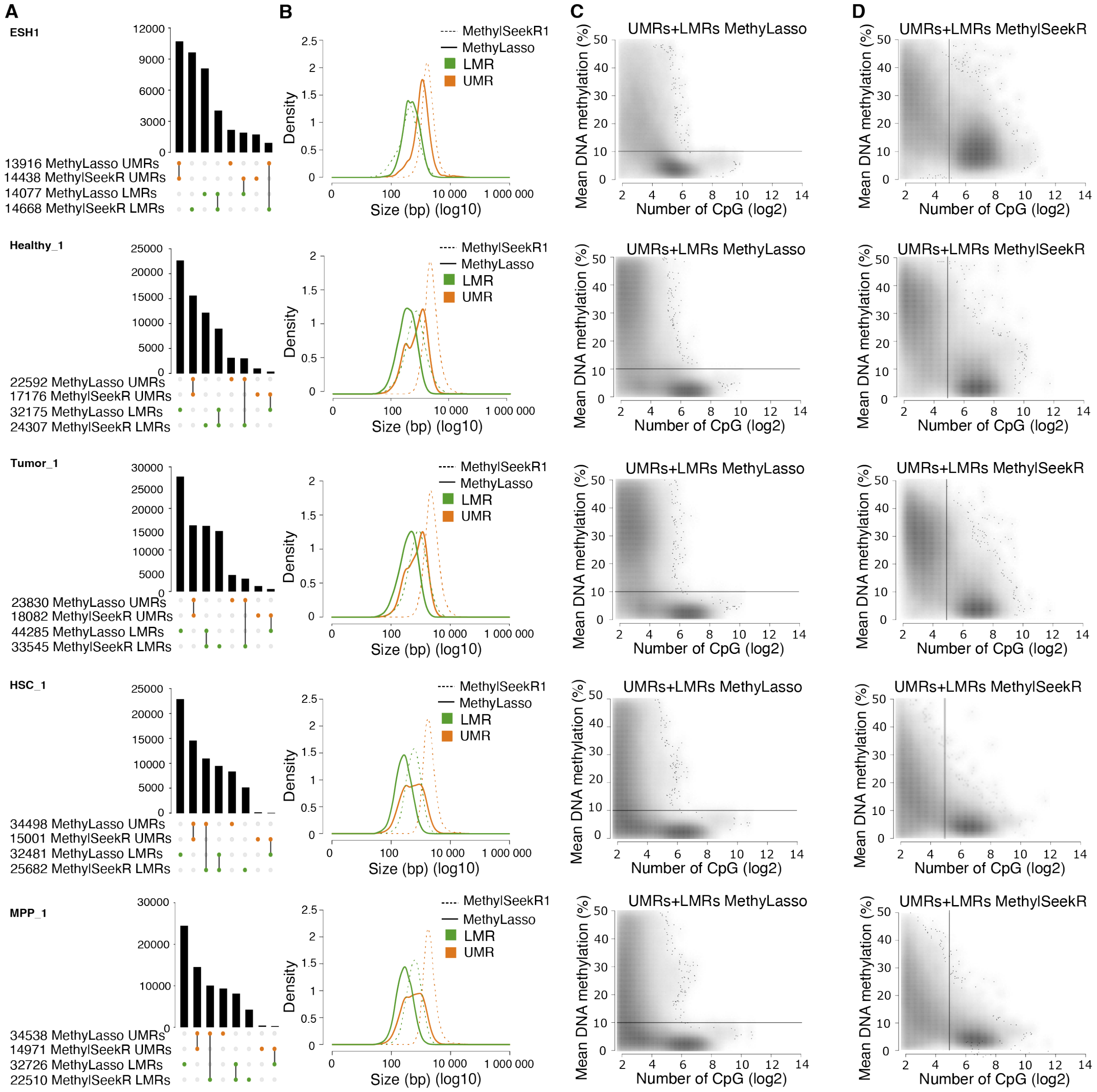

Supplementary Figure 2 - Features of unmethylated regions (UMRs) and low-methylated regions (LMRs)

Same as Figure 1B, C, F, G but for different datasets. ***A.*** *Upset plot of the number and overlap of UMRs and LMRs called by MethyLasso and MethylSeekR. MethylSeekR regions for replicate one are shown.* ***B.*** *Histogram of the size of the LMRs and UMRs called by MethyLasso and MethylSeekR.* ***C.*** *Density plot of the number of CpGs versus mean DNA methylation in each region from MethyLasso. The black line represents the MethyLasso threshold between LMRs (top) and UMRs (bottom).* ***D.*** *Density plot of the number of CpGs versus DNA methylation in each region from MethylSeekR. The black line represents the MethylSeekR threshold between LMRs (left) and UMRs (right).*

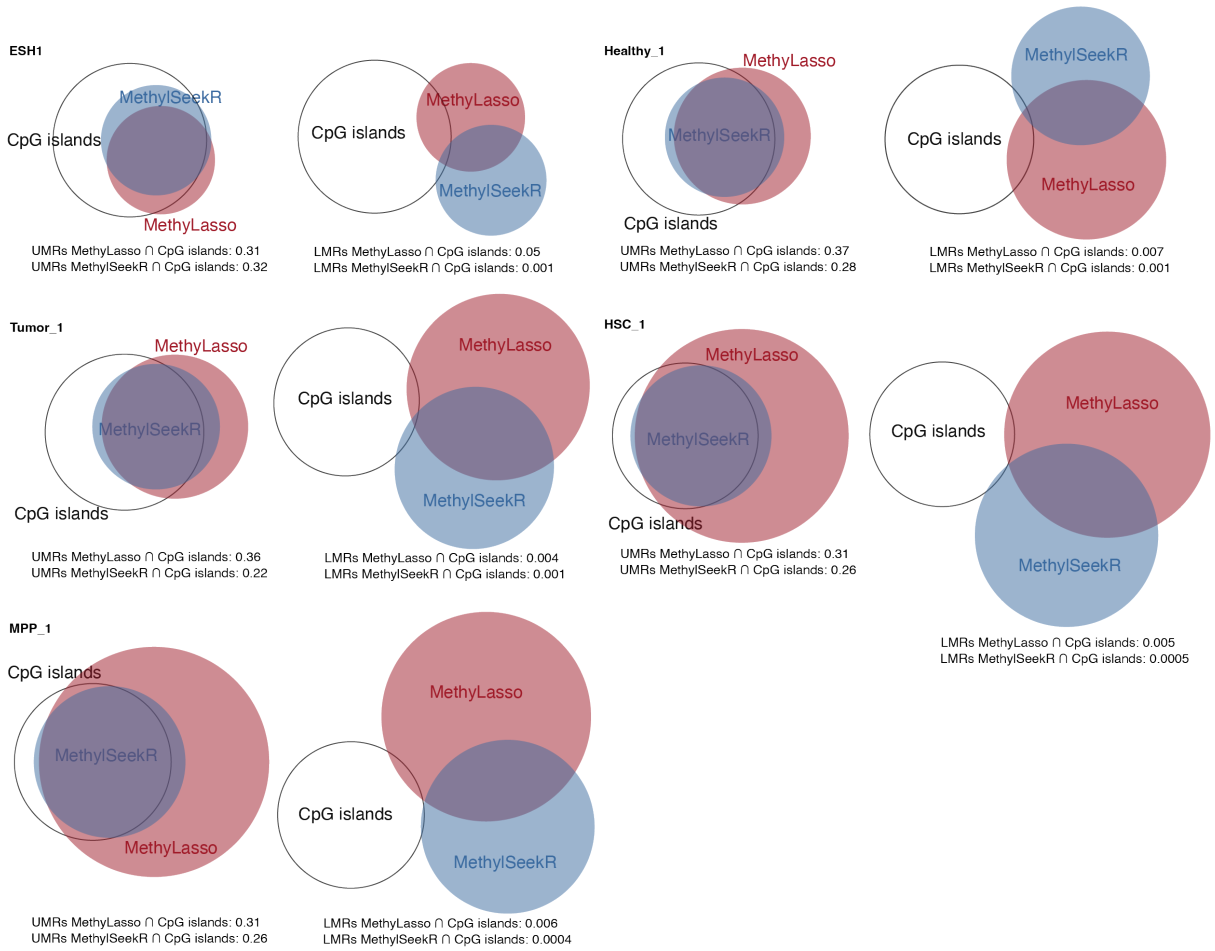

Supplementary Figure 3 - Overlap of UMRs and LMRs with CpG islands
Same as Figure 1D,E but for different datasets. Venn diagrams showing the overlap of CpG islands with LMRs or UMRs from MethyLasso and MethylSeekR.

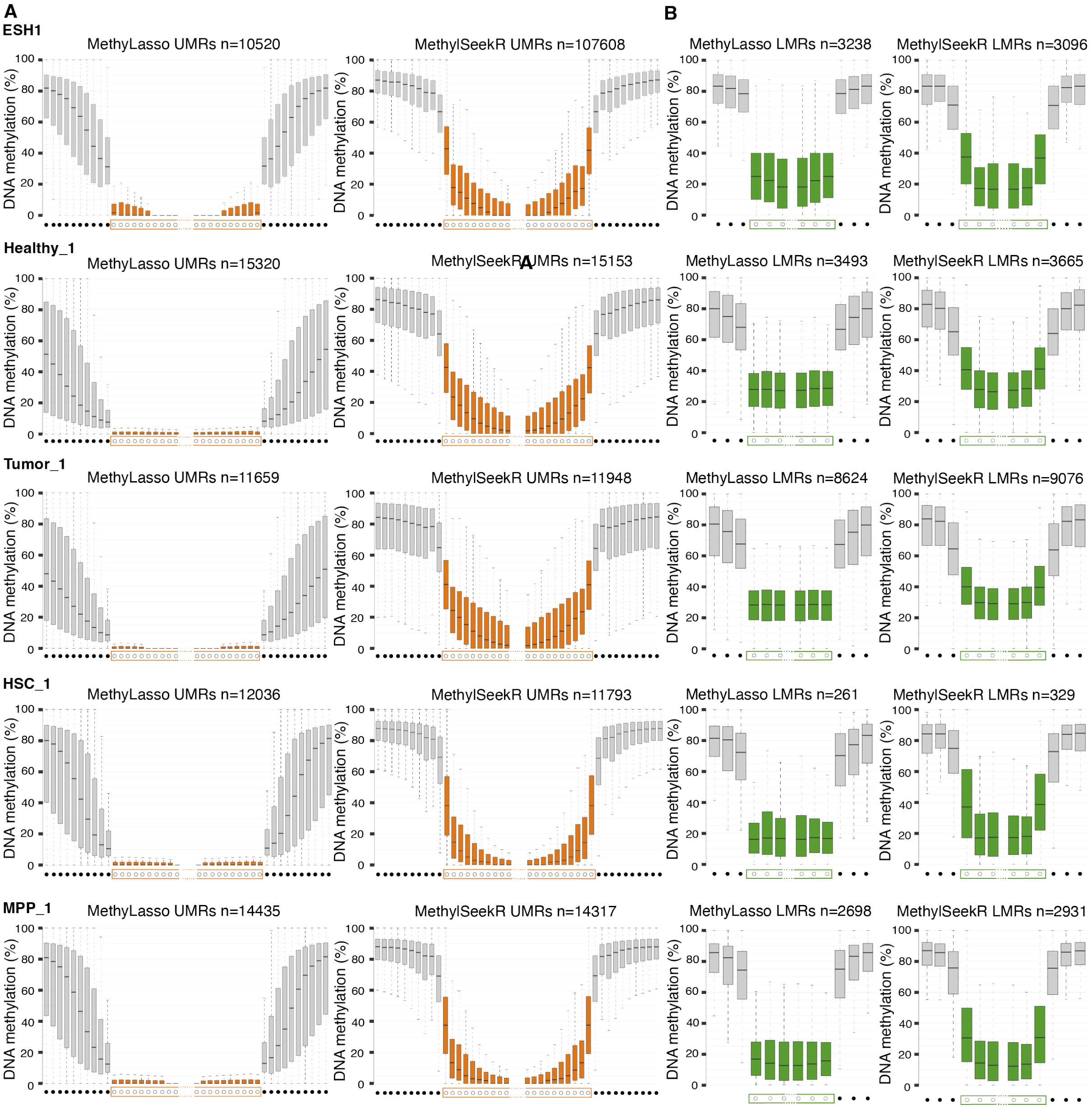

Supplementary Figure 4 - Boundaries of unmethylated regions (UMRs) and low-methylated regions (LMRs)

Same as Figure 1J and K but for different datasets. ***A.*** *Boxplot of the DNA methylation levels at individual CpGs at UMR boundaries called by MethyLasso and MethylSeekR. Grey boxes correspond to the ten CpGs upstream and downstream of the UMRs. Orange boxes correspond to the ten CpGs at the beginning or the end of the UMRs.* ***B.*** *Boxplot of the DNA methylation at individual CpGs at LMR boundaries called by MethyLasso and MethylSeekR. Grey boxes correspond to the three CpGs upstream and downstream of the LMRs. Green boxes correspond to the three CpGs at the beginning or the end of the LMRs.*

Supplementary Figure 5 - Features of partially methylated domains (PMDs) *(see next page)*

A,B,C,D. *Histogram of MethylSeekR alpha values. Samples contains PMDs if they have a bimodal or long-tailed distribution with an important proportion of α >1.* ***E,F,G,H.*** *Scatterplot showing for all MethyLasso segments their mean DNA methylation versus standard deviation. Segments with mean methylation below 70% and standard deviation above 0.15 are called as PMDs.* ***I,J,K,L,M.*** *Analyses for Colon healthy data.* ***N,O,P.*** *Analyses for Colon tumor data.* ***I,N.*** *Barplot showing the percent of the genome covered by PMDs called by different methods.* ***J,O.*** *Upset plot of the number and overlap of*

PMDs from the different methods. ***K,P.*** *Boxplot of the mean DNA methylation of the PMDs shared by all methods or only found by one or two methods. Corresponding Wilcoxon p-value.* ***L.*** *Genome browser view of an example of PMDs from DNMTools identified as a UMR by MethyLasso. Region from hg38 at chr12:12,660,000-12,790,000.* ***M.*** *Venn diagram showing the overlap of PMDs only identified by DNMTools with MethyLasso LMRs/UMRS/DMVs.*

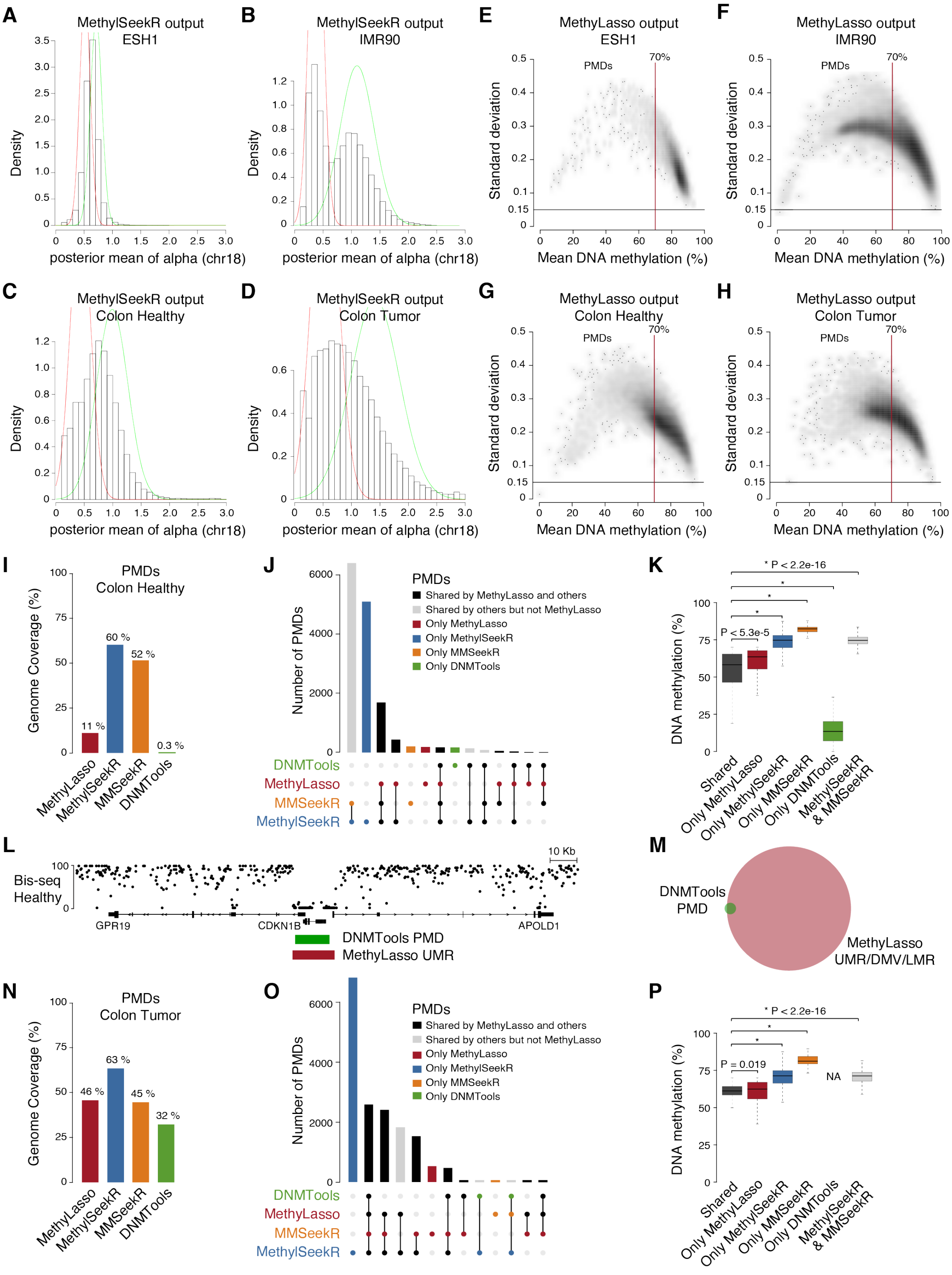

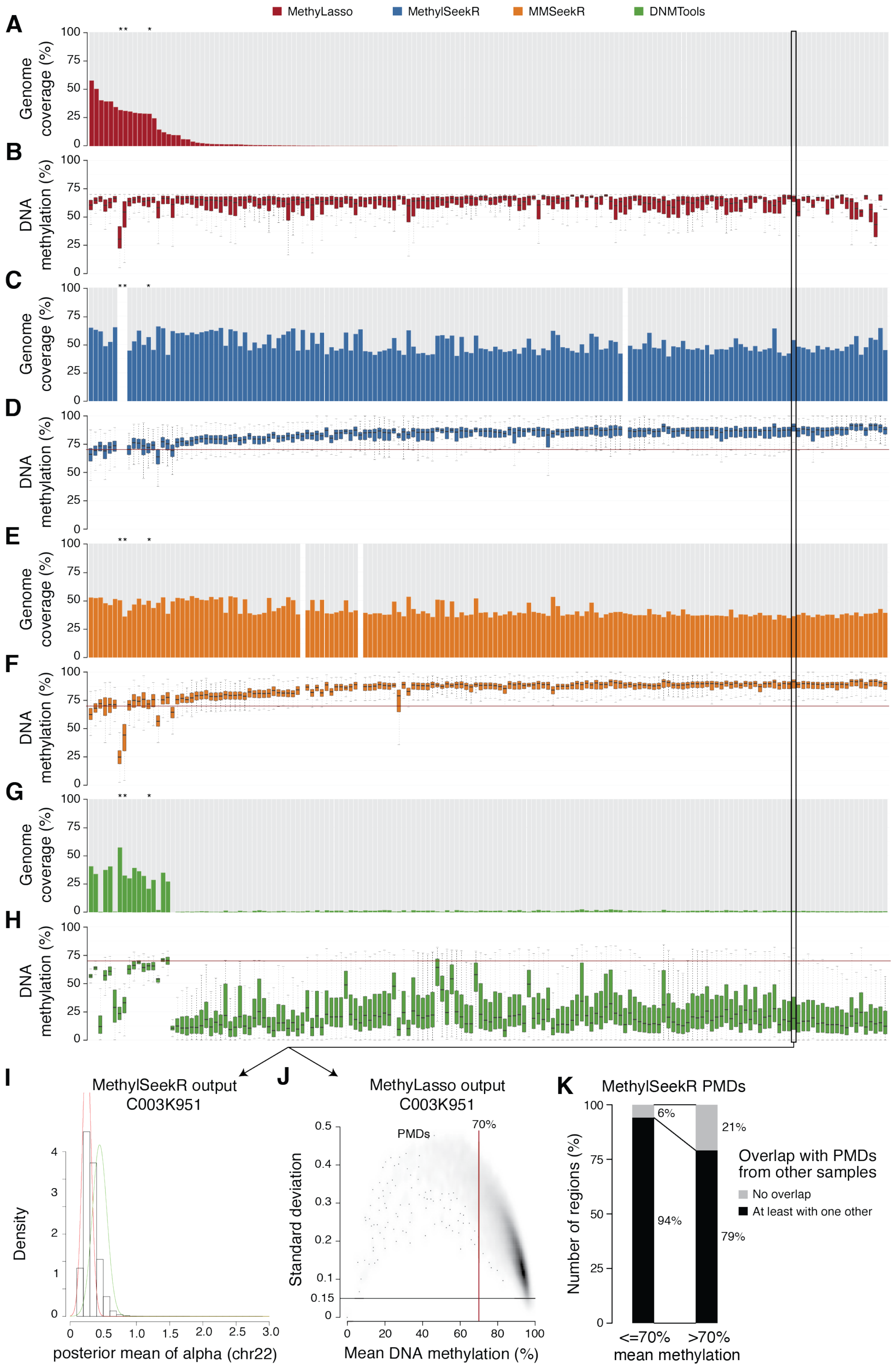

MethyLasso
MethylSeekR
MMSeekR
DNMTools
A
Genome coverage (%)
B
DNA methylation (%)
C
Genome coverage (%)
D
DNA methylation (%)
E
Genome coverage (%)
F
DNA methylation (%)
G
Genome coverage (%)
H
DNA methylation (%)
I
MethylSeekR output C003K951
Density
posterior mean of alpha (chr22)
J
MethyLasso output C003K951
PMDs
70%
Standard deviation
Mean DNA methylation (%)
K
MethylSeekR PMDs
Number of regions (%)
6%
21%
94%
79%
Overlap with PMDs from other samples
No overlap
At least with one other
<=70%
>70%
mean methylation

Supplementary Figure 6 - Analysis of 167 methylomes from Salhab et al. (14)

A,C,E,G*. Genome coverage of the PMDs identified using MethyLasso, MethylSeekR, MMSeekR and DNMTools respectively. Samples are ordered using the genome coverage of the MethyLasso PMDs.* ***B,D,F,H****. Boxplots showing the distribution of the methylation level of the PMDs.* ***I.*** *Example from sample C003K951 of the histogram of MethylSeekR alpha values. Samples contains PMDs if they have a bimodal or long-tailed distribution with an important proportion of α >1, which is not the case here.* ***J.*** *Example from sample C003K951 of the scatterplot showing for all MethyLasso segments their mean DNA methylation versus standard deviation.* ***K.*** *Proportion of MethylSeekR PMDs that have a mean methylation below or above 70% that overlap with PMDs from other samples. * denotes samples from cell lines (HepG2 and HepaRG) or cancer (G203). Samples from left to right in panels A-H are: G202, bmPCs-V156, 63349.DEEP.51_Hf03_BlEM_Ct, 63077.DEEP.41_Hf02_LiHe_Ct, S00UJK51, csMBC_NC11_41, 62967.DEEP.01_HepG2_LiHG_Ct1, 63003.DEEP.01_HepaRG_LiHR_D31, E098, S01GU951, S0164R51, 63098.DEEP.41_Hf03_LiHe_Ct, G203, 63052.DEEP.41_Hf01_LiHe_Ct, 63462.DEEP.51_Hf05_BlTA_Ct, S014WG51, E066, 63405.DEEP.51_Hf04_BlEM_Ct, S014QS51, 63382.DEEP.51_Hf04_BlCM_Ct, PC_T13_1, S00XTP51, 63340.DEEP.51_Hf03_BlCM_Ct, PC_T14_10, S017RE51, C00256A3bs, C003JB51, S004P151, E105, S001U352, S002ND51, E104, PC_T12_20, S00DCS53, E094, E095, E079, S014QS55, S00DCS51, E113, E109, C005UI52, G201, S00BJM52, GC_T14_11, 63118.DEEP.43_Hm01_BlMo_Ct, C003N351, S00V6553, C003VO56, E112, E106, 63156.DEEP.43_Hm03_BlMa_TE, 63194.DEEP.43_Hm03_BlMo_Ct, P581, 63137.DEEP.43_Hm03_BlMa_Ct, 63360.DEEP.51_Hf03_BlTN_Ct, GC_T14_10, 63175.DEEP.43_Hm03_BlMa_TO, C000S5A2bs, 63236.DEEP.43_Hm05_BlMa_TE, S01E03A1, S00TU2A1, S00TXXA1, P583, S006VI53, S00TV0A1, S006YC51, 63434.DEEP.51_Hf04_BlTN_Ct, 63216.DEEP.43_Hm05_BlMa_Ct, 63276.DEEP.43_Hm05_BlMo_Ct, 63256.DEEP.43_Hm05_BlMa_TO, S00TWZA1, P586, P584, S00U0LA1, P585, S00TYVA1, S01MAPA1, S0039051, S00E8W51, P582, C004GD51, C000S5A1bs, S00H6O51, C0010KA2bs, C0010KA1bs, S00BJM51, S010NDA1, S001S751, S010R5A1, S00K88A1, P580, S000RD54, NECH0001_2, C001UYA3bs, S001S753, S001U353, C002CTA1bs, S00JFZA1, S0062252, C001UYA1bs, S01G2SA1, C005PS51, S010Q7A1, C00256A1bs, S010S3A1, S00FTN51, C004SQ51, S01ECGA1, NECH0001_3, NECH0001_1, S00K7AA1, S01BAWA1, C0011IA3bs, S00TC1A1, S00VDSA1, C006G551, S006XE53, S00JHVA1, C0068L51, C003VO55, S00VEQA1, S00JGXA1, C0067N51, S00FWHA1, S01B63A1, S01B55A1, S01B71A1, S01B9YA1, S0022I53, S01BHIA1, S01E8O51, S01BFMA1, S00JKPA1, S01BEOA1, S01BGKA1, S01B47A1, S00DVR51, S01BBUA1, S01BCSA1, C0066P51, S001MJ51, S00D7151, S00BS451, C003K951, S00VFOA1, C00184A3bs, S00V49N1, C005VG51, S00V2DN1, S0022I51, S00JMLA1, S00FXFA1, S00V3BN1, S007SK51, S00BHQ51, S008H151, S007G756, S00C2F51, S00JNJA1, S009W451, S007DD51, S00DM851, S0018A52*

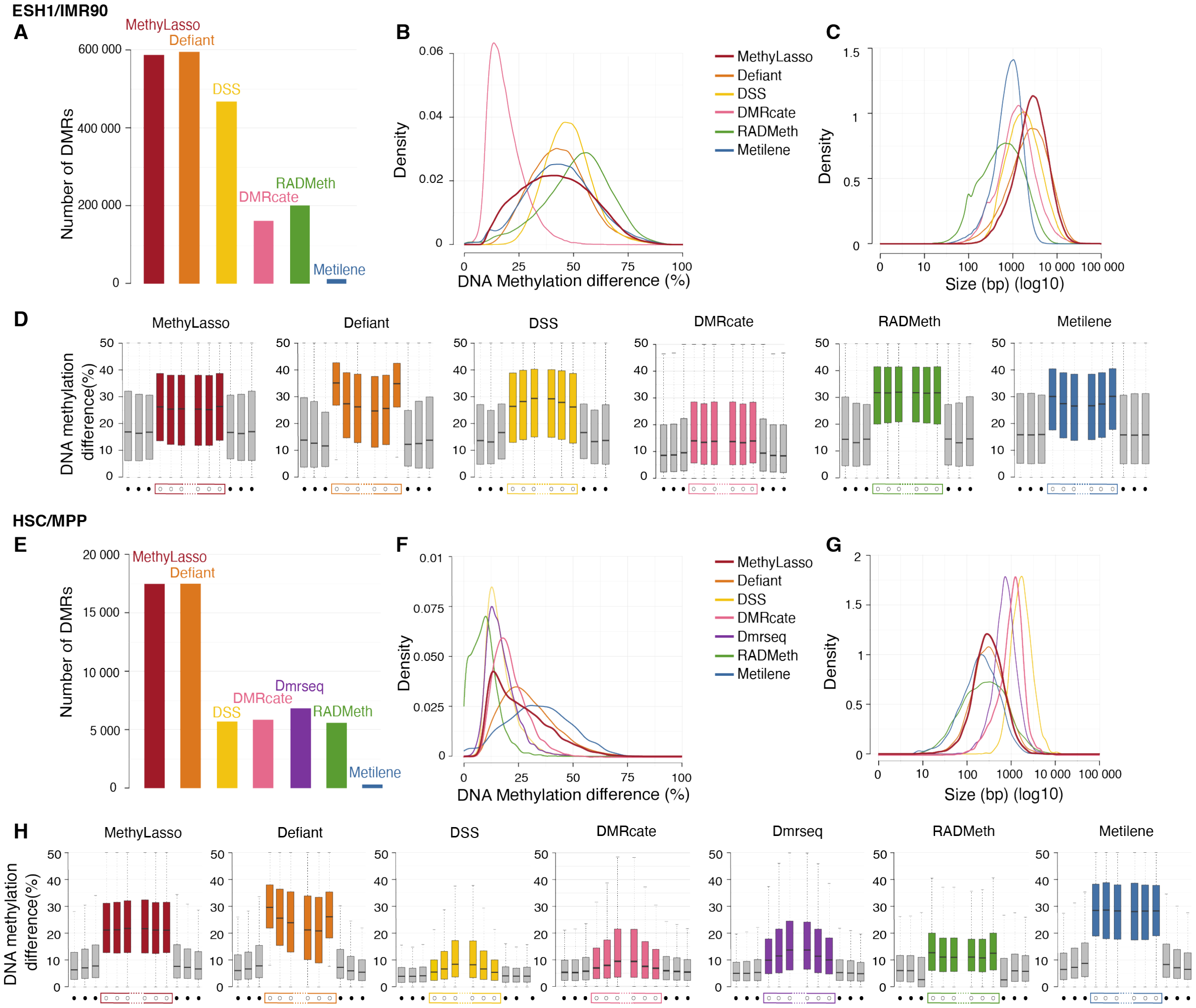

Supplementary Figure 7 - Features of differentially methylated regions (DMRs)

*Same as Figure 3 but for the best 270,000 DMRs for ESH1 vs. IMR90 (**A-D**) and the best 5,000 DMRs for HSC vs. MPP (**E-H**). **A&E.** Barplot of the number of DMRs identified by the different methods. **B&F.** Histogram of the absolute DNA methylation difference in the best DMRs called by the different methods. **C&G.** Histogram of the size of the best called by the different methods. **D&H.** Boxplot of the difference of DNA methylation at individual CpGs at DMR region boundaries called by the different methods. Grey boxes correspond to the three CpGs upstream and downstream of the DMRs. Colored boxes correspond to the three CpGs at the beginning or the end of the DMRs. Note that DMRs could not be identified by Dmrseq for ESH1 vs IMR90 data since it had no replicate for the ESH1 condition.*

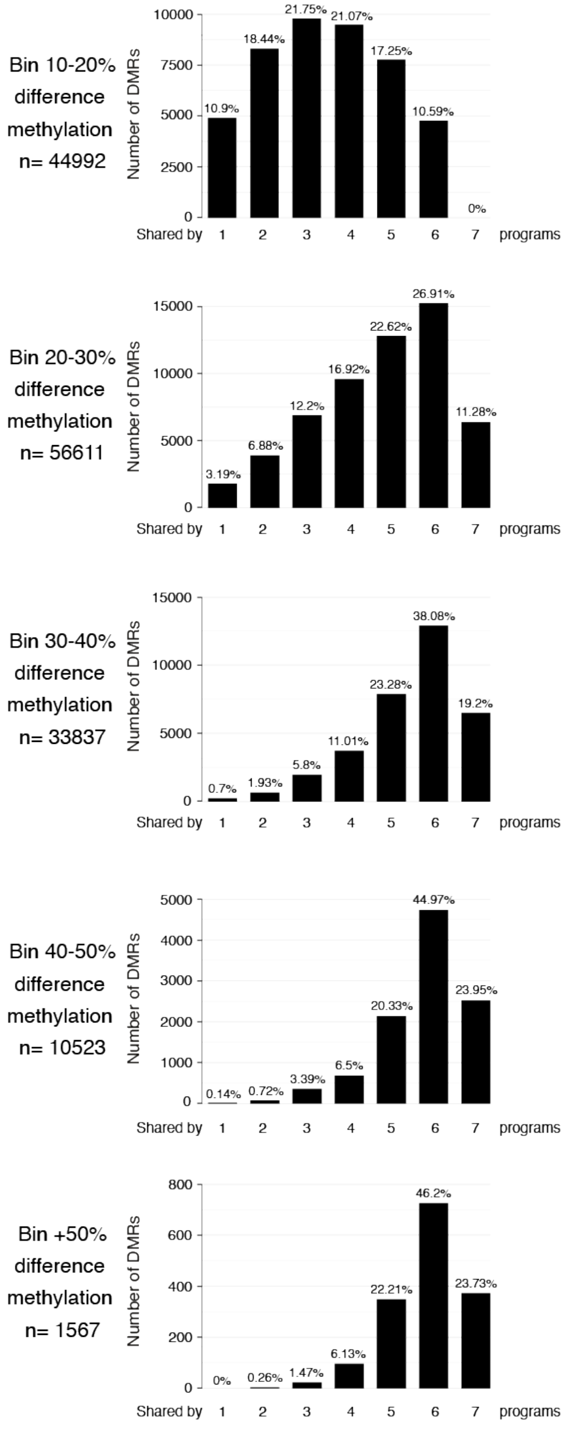

Supplementary Figure 8 - Consistency between DMRs from different approaches at different methylation difference levels

Barplots summing the number of regions identified by all, some or none of the methods for different bins of methylation difference. Data from human colon cancer compared to healthy samples.

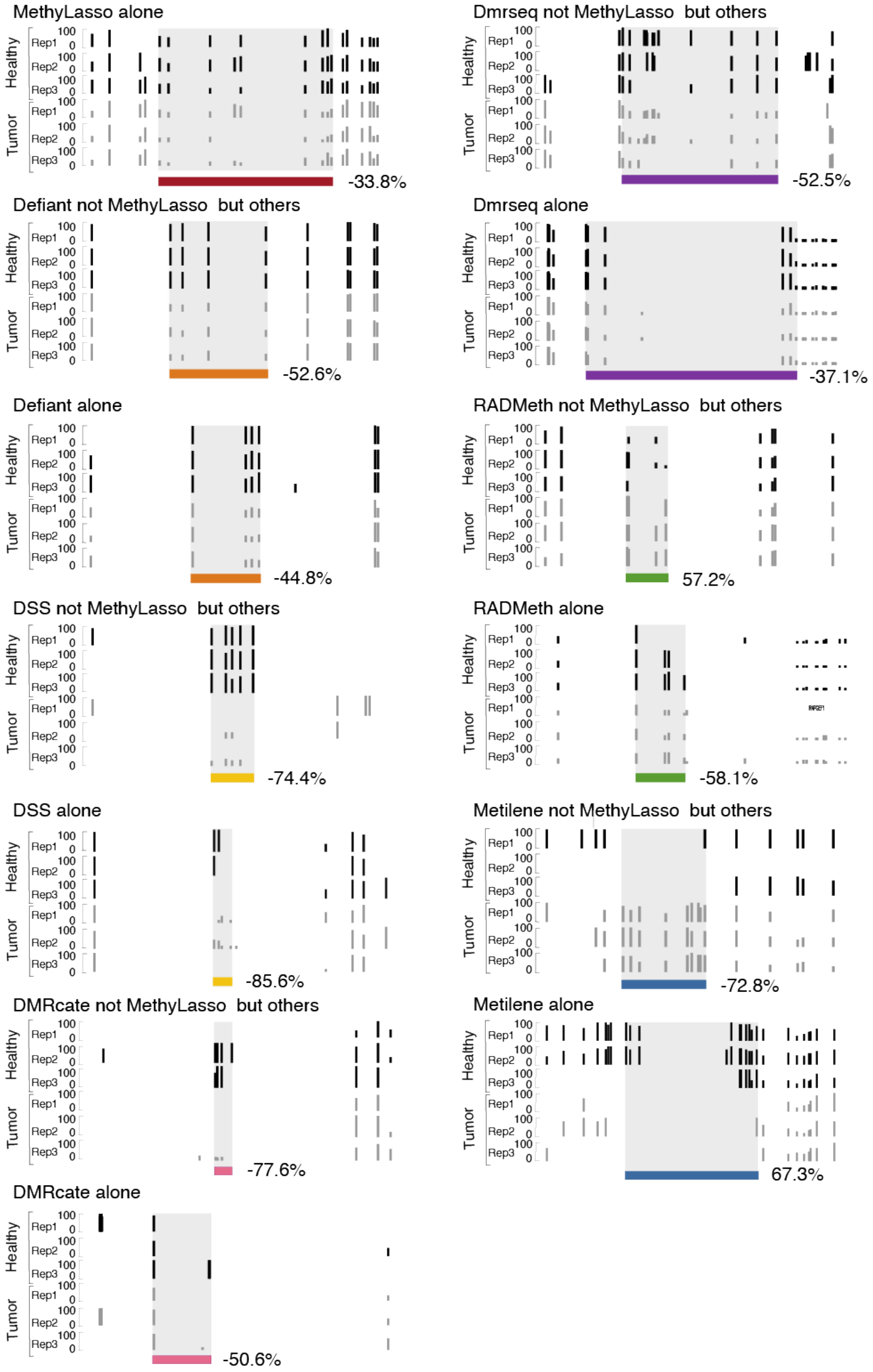

Supplementary Figure 9 - Example of DMRs from the different methods

Examples of DMRs from each category in Figure 4C chosen among the top ten.

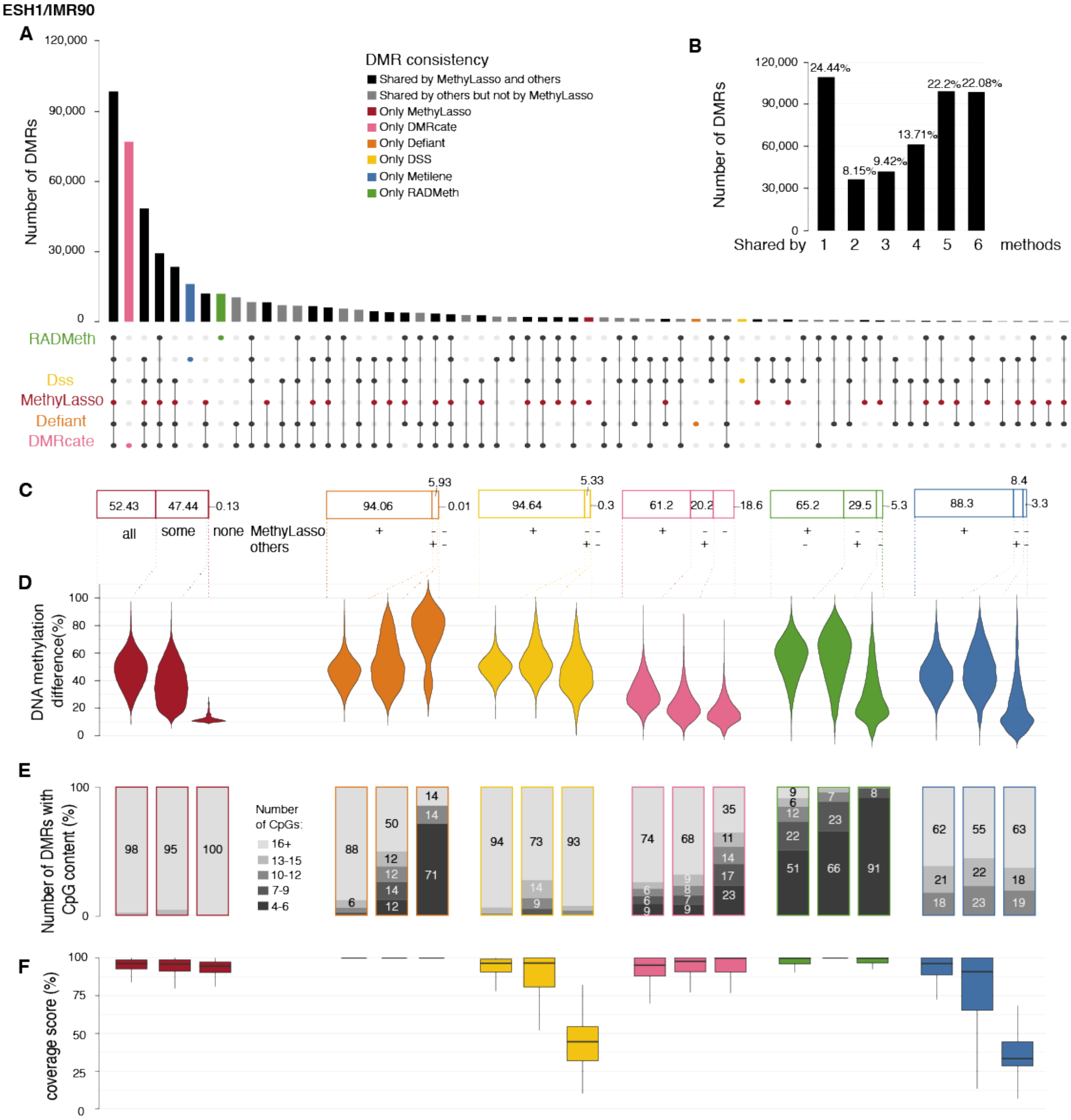

Supplementary Figure 10 - Consistency between DMRs from different approaches in ESH1 vs. IMR90 data

Same as Figure 4. ***A.*** *Upset plot of the overlap of best 270,000 DMRs called by the different methods. Bars corresponding to DMRs identified by MethyLasso are represented in red.* ***B.*** *Barplot summarizing the upset plot by summing the number of regions identified by all, some or none of the methods.* ***C.*** *Cumulative barplot showing the percent of best 100,000 MethyLasso DMRs overlapping with all others 270,000 DMRs, some others or none of the other methods. For other methods, percent of their best 100,000 DMRs overlapping with 270,000 DMRs from MethyLasso (+), not MethyLasso but others (-/+) or not MethyLasso nor others (-/-).* ***D.*** *Absolute DNA methylation difference in the DMRs from the categories in C.* ***E.*** *Cumulative barplot showing the number of CpGs in the DMRs from the categories in C.* ***F.*** *Boxplot showing the coverage of CpGs in the DMRs from the categories in C.*

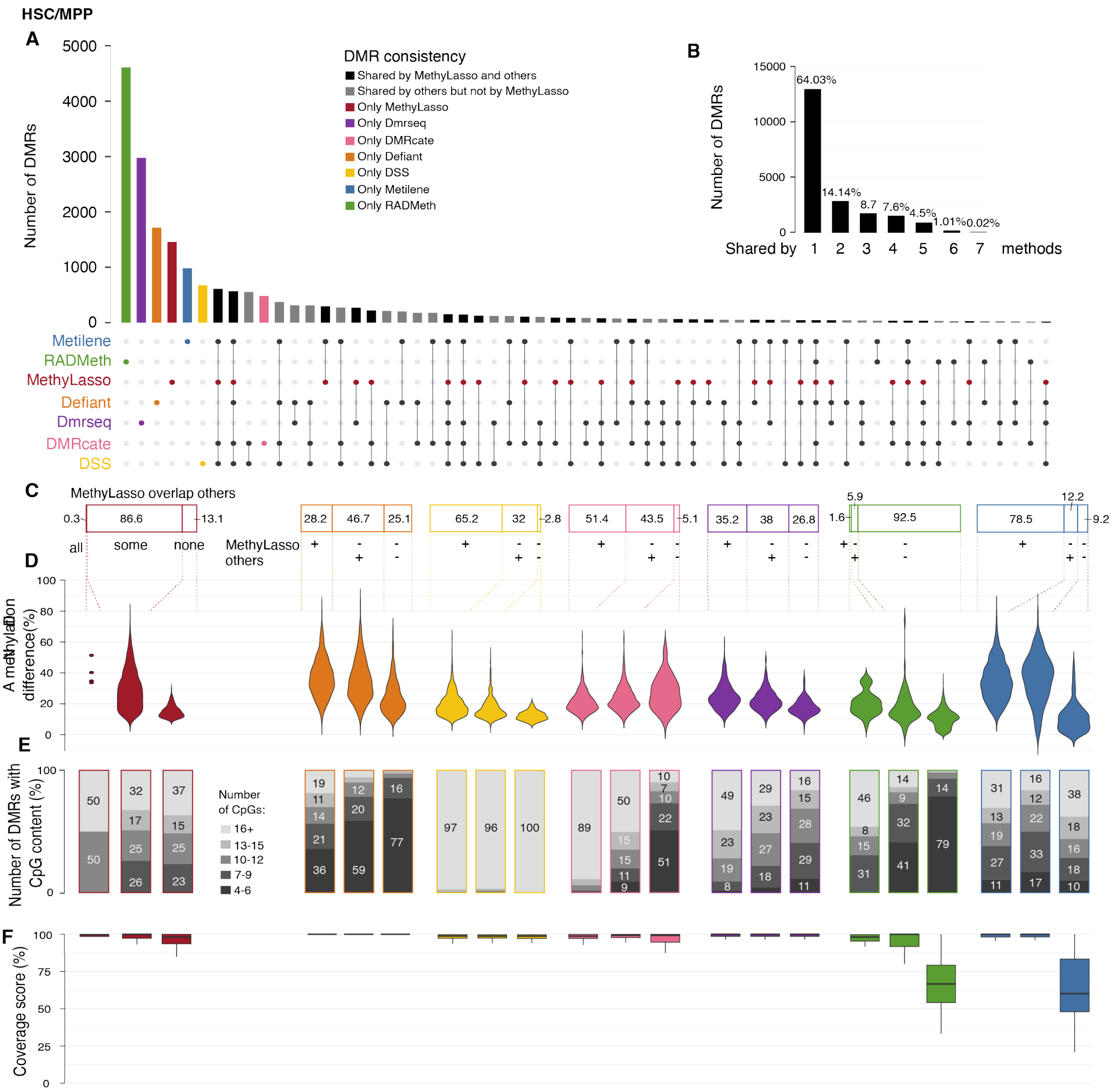

Supplementary Figure 11 - Consistency between DMRs from different approaches in HSC vs. MPP data

Same as Figure 4. ***A.*** *Upset plot of the overlap of best 5,000 DMRs called by the different methods. Bars corresponding to DMRs identified by MethyLasso are represented in red.* ***B.*** *Barplot summarizing the upset plot by summing the number of regions identified by all, some or none of the methods.* ***C.*** *Cumulative barplot showing the percent of best 1,600 MethyLasso DMRs overlapping with all others 5,000 DMRs, some others or none of the other methods. For other methods, percent of their best 1,600 DMRs overlapping with 5,000 DMRs from MethyLasso (+), not MethyLasso but others (-/+) or not MethyLasso nor others (-/-).* ***D.*** *Absolute DNA methylation difference in the DMRs from the categories in C.* ***E.*** *Cumulative barplot showing the number of CpGs in the DMRs from the categories in C.* ***F.*** *Boxplot showing the coverage of CpGs in the DMRs from the categories in C.*

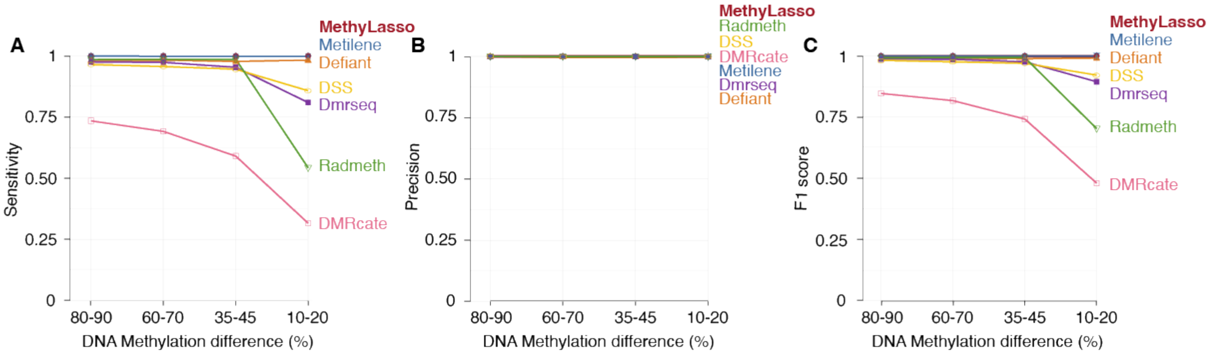

Supplementary Figure 12 - Sensitivity and precision of the method using simulated DMRs

Simulated DMRs in different bins of DNA methylation difference from Metilene using a homogeneous background. ***A.*** *Sensitivity or recall of the predictions measured as the number of true positives among all simulated.* ***B.*** *Precision of the predictions measured as the number of true positives among all predicted.* ***C.*** *F1 score or accuracy of the predictions measured using both the sensitivity and precision.*
